# The Prevalence of *Coxiella burnetii* in Hard Ticks in Europe and Their Role in Q Fever Transmission Revisited—A Systematic Review

**DOI:** 10.3389/fvets.2021.655715

**Published:** 2021-04-26

**Authors:** Sophia Körner, Gustavo R. Makert, Sebastian Ulbert, Martin Pfeffer, Katja Mertens-Scholz

**Affiliations:** ^1^Institute of Bacterial Infections and Zoonoses (IBIZ), Friedrich-Loeffler-Institute, Federal Research Institute for Animal Health, Jena, Germany; ^2^Department of Immunology, Fraunhofer Institute for Cell Therapy and Immunology, Leipzig, Germany; ^3^Institute of Animal Hygiene and Veterinary Public Health, University of Leipzig, Leipzig, Germany

**Keywords:** ticks, Coxiella, prevalence, molecular detection, Coxiella-like, endosymbionts, vector

## Abstract

The zoonosis Q fever is caused by the obligate intracellular bacterium *Coxiella burnetii*. Besides the main transmission route *via* inhalation of contaminated aerosols, ticks are discussed as vectors since the first isolation of the pathogen from a *Dermacentor andersonii* tick. The rare detection of *C. burnetii* in ticks and the difficult differentiation of *C. burnetii* from *Coxiella*-like endosymbionts (CLEs) are questioning the relevance of ticks in the epidemiology of Q fever. In this review, literature databases were systematically searched for recent prevalence studies concerning *C. burnetii* in ticks in Europe and experimental studies evaluating the vector competence of tick species. A total of 72 prevalence studies were included and evaluated regarding DNA detection methods and collection methods, country, and tested tick species. Specimens of more than 25 different tick species were collected in 23 European countries. Overall, an average prevalence of 4.8% was determined. However, in half of the studies, no *Coxiella*-DNA was detected. In Southern European countries, a significantly higher prevalence was observed, possibly related to the abundance of different tick species here, namely *Hyalomma* spp. and *Rhipicephalus* spp. In comparison, a similar proportion of studies used ticks sampled by flagging and dragging or tick collection from animals, under 30% of the total tick samples derived from the latter. There was no significant difference in the various target genes used for the molecular test. In most of the studies, no distinction was made between *C. burnetii* and CLEs. The application of specific detection methods and the confirmation of positive results are crucial to determine the role of ticks in Q fever transmission. Only two studies were available, which assessed the vector competence of ticks for *C. burnetii* in the last 20 years, demonstrating the need for further research.

## Introduction

*Coxiella burnetii* as the causative agent of the zoonosis Q fever is distributed worldwide, except New Zealand. This infectious disease may have a significant impact on animal welfare, human health, and economies (1, 2). *Coxiella burnetii* is a gram-negative bacterium that replicates as an obligate intracellular pathogen under acidic and microaerophilic conditions in phagolysosome-like compartments of cells, predominantly macrophages (3). Infection of hosts mainly occurs due to inhalation of contaminated dust and aerosols (4). During infection, *C. burnetii* shows a tropism for the reproductive tissue and the mammary gland. Therefore, *C. burnetii* is primarily shed during parturition and *via* the milk (5). The considered main reservoirs for human infections are domestic ruminants, whereas other species, such as dogs or horses, can play a minor role as carriers (6). In their birth products, large amounts of infectious agents can be detected (7, 8). As the infective dose by inhalation is <10 bacteria, farmers, and veterinarians are especially at high risk of acquiring this disease through contact with infected animals and their products (9, 10). *Coxiella burnetii* develops spore-like forms, which are resistant to environmental stressors such as desiccation or sunlight. Therefore, the bacteria survive under adverse conditions over long periods in the soil or other dry substances (11).

An infection in ruminants, also termed coxiellosis, is often asymptomatic and not noticed until human Q fever cases occur (12). Decrease in fertility and increase of abortion and stillbirth are common indications of an ongoing Q fever disease in herds or flocks of ruminants (13, 14).

Outbreaks of Q fever, as seen in the Netherlands in 2007–2010, clearly show the huge impact of this infectious disease not only on agricultural economics but also on human health (15, 16). Acute human Q fever infection presents mainly flu-like symptoms with possible complications concerning the lung and liver. Although 60% of acute cases remain asymptomatic, in 1–5% of cases, chronic Q fever may develop (17, 18). This often affects the heart valves as endocarditis. Antibiotic treatment is mandatory over months and, in some cases, up to several years (17–19). Besides the long and difficult treatment, the disease can also be fatal in acute and chronic cases. Although the mortality of acute Q fever was assessed to be 1% in a study on hospitalized patients, the mortality in chronic cases has been reported as 13% and even up to 38% during the Dutch outbreak mentioned earlier (1, 18, 20).

The bacterium is known as an infectious agent since the 1930s when it caused an outbreak of query fever among abattoir workers. In the years after its first isolation from a *Dermacentor andersonii* tick (21), early experimental studies showed horizontal transmission of the agent from ticks to mammalian hosts and suggested that several tick species, e.g., *Haemaphysalis humerosa, Haemaphysalis bispinosa*, and *Rhipicephalus sanguineus*, may be capable of transmitting *C. burnetii* (22–25). Although transstadial transmission was shown for most of the examined tick species, the transovarial transmission was reported rarely (25, 26). Later, it was demonstrated that *Hyalomma dromedarii* infected by inoculation excreted *C. burnetii* with their saliva (27). Duron et al. summarized the vector competence data for seven hard tick species (28). Hence, since its first detection, *C. burnetii* has been discussed as a ruminant-associated tick-borne bacterium.

During outbreaks of Q fever, ticks are regularly tested for *C. burnetii*. Until today, no *C. burnetii*-positive ticks associated with outbreaks have been documented. Human infection after tick exposure has been reported, but due to the predominant infection route by inhalation, the source of infection remains unclear (29, 30).

Besides pathogen transmission, ticks are host to many endosymbionts and harbor a diverse microbiome (31). Although *C. burnetii* was the only known member of the genus *Coxiella* for a long time, closely related bacteria were found in recent studies, referred to as *Coxiella*-like endosymbionts (CLEs) (32, 33). This heterogenic group of bacteria with different genome sizes and gene content shows up to 97% genome identity with *C. burnetii* (33–37). Genetically, CLEs are classified into four clades (A–D), and *C. burnetii* belongs to clade A, which otherwise contains CLE associated with soft ticks (38).

*Coxiella*-like endosymbionts were detected in many different tick species, and for some species, an obligatory mutualism was proven (39–41). As obligate hematophagous arthropods, ticks are dependent on external vitamin B synthesis, likely supplied by the harbored bacteria (40, 42). Furthermore, a positive effect on fecundity was shown for *R. sanguineus*. Treatment with ofloxacin resulted in a significantly lower egg mass, hatching rate, and viability of larvae in this tick species (39). Similar results were shown for *Haemaphysalis longicornis, Rhipicephalus microplus*, and *Rhipicephalus haemaphysaloides*, which were treated with tetracycline and kanamycin, respectively, indicating the influence of CLE on reproduction (43–45).

CLEs seem to be apathogenic, but single studies showed pathogenic potential in particular cases. Fatal infection with CLE was occasionally observed in different bird species (46–48). Furthermore, a skin-associated inflammation caused by the tick-borne bacterium *Candidatus Coxiella massiliensis* was described (49).

The polymerase chain reaction was established in the 1980s and was subsequently used as the predominant detection method. Until then, ticks were primarily tested for the occurrence of *C. burnetii* and related pathogens with staining methods and animal infection experiments (50–52). Because *C. burnetii* is difficult to isolate and cultivate as an obligate intracellular organism, molecular diagnostic using PCR is the method of choice for its detection. Various protocols target several plasmids and chromosomal genes, for example, IS1111, *icd, com1, sodB*, and GroEL/*htpB* (53–55). The target gene IS1111 is a transposase-like insertion sequence, and the amount of copies from 7 to 110 per genome varies between isolates. Thus, the use for quantification is limited, but due to the higher number of targets per bacterium, this signal amplification leads to a higher sensitivity compared with single-copy targets (53). In contrast, *icd*, encoding for the isocitrate dehydrogenase gene, and *com1*, encoding for the *C. burnetii* outer membrane protein 1, are single-copy genes allowing quantification (53, 54). The commonly used target genes for *C. burnetii* cross-react with CLE and may lead to misidentification (28, 56). In a study by Duron, roughly one-third of CLE-positive ticks were positive for the IS1111 element (57). Furthermore, IS1111 was shown to be the most unspecific marker for detection of *C. burnetii* in CLE-positive ticks, followed by GroEL/*htpAB* (for chaperone heat shock protein), whereas *icd* was not amplified in the samples (56). However, the *icd* sequence of a novel CLE derived from *Carios capensis* soft ticks was >90% similar to *C. burnetii* (58). As a consequence, usage of a target gene of low specificity could lead to an overestimation of the role of ticks as host and vector of *C. burnetii*. To distinguish between *C. burnetii* and CLE, there is no specific method available, owing to the fact that the group of CLE is very heterogeneous. Therefore, the most reliable method is the sequencing of PCR-positive samples using highly conserved genes such as *rrs* or *groEL* (56, 59).

Ticks (Ixodida) are obligate blood-feeding ectoparasites with a global distribution. A total of 67 tick species are reported in Europe and Northern Africa (60). Out of these, only a small number, i.e., 15–17 hard tick species, depending on their confirmed taxonomic status as a distinct species or subspecies, have been found to harbor either *C. burnetii* or CLE (28, 61). When considering a particular tick species to serve as a vector for a given pathogen, certain aspects of the tick's life cycle should be considered in addition to the sole finding of molecular traces or even morphological structures within an examined tick. This information will help to understand further whether this tick species has vector competence, defined as the capability to transmit a given pathogen horizontally or vertically, which is an essential aspect of the vector capacity. The latter is more relevant for determining the infection risk, as it includes parameters such as longevity, feeding behavior (duration, frequency, and preferred blood source), population density, and frequency of host contact encounters. In this regard, ticks, which are feeding on different hosts in every life stage (three-host-ticks) with a large geographical range, high population densities, and promiscuous feeding behavior are relevant, when it comes to pathogen transmission. The most common hard tick of Europe, the castor bean tick *Ixodes ricinus*, is fulfilling these criteria, making it the most important vector tick species for bacterial and viral tick-borne diseases, e.g., *Borrelia burgdorferi* s.l. or tick-borne encephalitis virus, in Europe (62). Such vector-borne agents, some with a wide range of host species, may have their reservoir in wildlife, leading to a sylvatic transmission cycle between arthropods and wild animals. In addition, transmission can also occur in an urban or domestic cycle, including pets, livestock, or humans. Although *I. ricinus* is ubiquitous in Europe, other relevant tick species are not, and only for some geo-referenced distribution maps are available, e.g., for both *Dermacentor* species (63). However, the geographical distribution of a particular tick species is not static but rather subject to permanent habitat changes due to increased movement of humans, animals, and goods and, very importantly, the increasing ambient temperatures as major drivers. The high frequency of recent introductions of *Hyalomma* spp. ticks into temperate Europe is impressive sign of a very menacing trend of potent vector tick species moving into new areas (64, 65).

Recent prevalence studies using molecular methods aim at determining the percentage of ticks carrying *C. burnetii* among other diverse tick-borne pathogens. This allows surveillance of *C. burnetii* in ticks and benefits the identification of a potential risk of acquiring a Q fever infection by a tick-bite for humans or domestic animals.

Different methods exist for the sampling of ticks. As most European hard ticks seek hosts by questing on the vegetation and naturally intend to be wiped up by a passing host, the mainly used collection method is flagging or dragging. Using this method, a cotton blanket is dragged or flagged over the vegetation, considering that both methods favor different tick species (66). Ticks are stripped off and are hindered in their motion by the weave. The success of this method depends on the texture of the blanket and the structure of the vegetation (67). Activity peaks of most questing ticks are in spring and autumn. However, other studies observed a trend to a unimodal peak with the highest activity in the spring months (68, 69). Ticks can also be removed directly from their host. This method is used to examine the burden of pathogens associated with these animals and for the sampling of hunting ticks, such as *Hyalomma* spp., which are less likely to be collected by flagging (70).

This review gives a comprehensive overview of the present literature on *C. burnetii* in ticks and discusses the vector competence of European tick species for the Q fever agent. To this end, data of prevalence studies conducted in Europe concerning the occurrence of *C. burnetii* in ticks were systematically compiled and analyzed regarding the spatial occurrence, molecular detection method used, and the frequency of detection per tick species, as well as the detection of CLE. Furthermore, experimental approaches to investigate the vector competence of tick species for *C. burnetii* were collected. The determination of the vector competence of ticks is important to assess the role of ticks as a reservoir in Q fever epidemiology.

## Materials and Methods

Studies about *C. burnetii* in ticks were classified into two different categories. One group investigates the prevalence of ticks for *C. burnetii* in Europe. The other group discusses the vector competence of ticks based on experimental approaches on ticks infected under laboratory conditions. For identification of studies, the databases Pubmed and Web of Science were systematically searched for articles in English, published between 2000 and 2020. Search terms were “*coxiella* tick,” “*coxiella*-like,” “*coxiella* vector competence.” The search was completed on October 27, 2020. Additional studies were included using Google Scholar. References were sorted by the use of Endnote X7 (Clarivate Analytics, Philadelphia, USA). Only studies using DNA detection methods for *Coxiella* spp. on hard ticks obtained in Europe were included as prevalence studies. The definition of Europe used in this study refers to a geographical demarcation as von Strahlenberg's, including all countries of which the major part is situated between the Urals and the Bosphorus. Regions were defined according to the United Nations geoscheme for Europe in Southern Europe, Eastern Europe, Northern Europe, and Western Europe (71).

Further criteria for eligibility as prevalence studies were stated information of the number of tested ticks, the number of *Coxiella*-positive ticks, or amount and description of tick pools and at a minimum description of the tick genus or species name.

Data were extracted regarding information about the country in which the ticks were obtained. Furthermore, the number of tested and *Coxiella*-positive ticks and tick pools and the collection and detection method were included in the analysis. All publications were read, and data were extracted by two persons independently.

The proportion of positive ticks was calculated as the ratio of positive ticks to the total number of tested ticks. Prevalence was defined as the proportion of positive ticks multiplied by 100. When ticks were tested in pools, the proportion was estimated as minimum infection rate (MIR) using the formula

$$\begin{array}{l} \text{MIR}=\frac{\text{number of positive pools}}{\text{number of tested specimen}} \end{array}$$

Studies were evaluated, and confidence interval was calculated using Excel 2016 (Microsoft Corporation, Redmond, USA). For statistical analysis, the Kruskal–Wallis test and Mann–Whitney U test for data lacking normal distribution were performed using SPSS V.22 (IBM, Armonk, USA), and results were considered significant in the case of *p* < 0.05.

## Results

**Figure 1:**
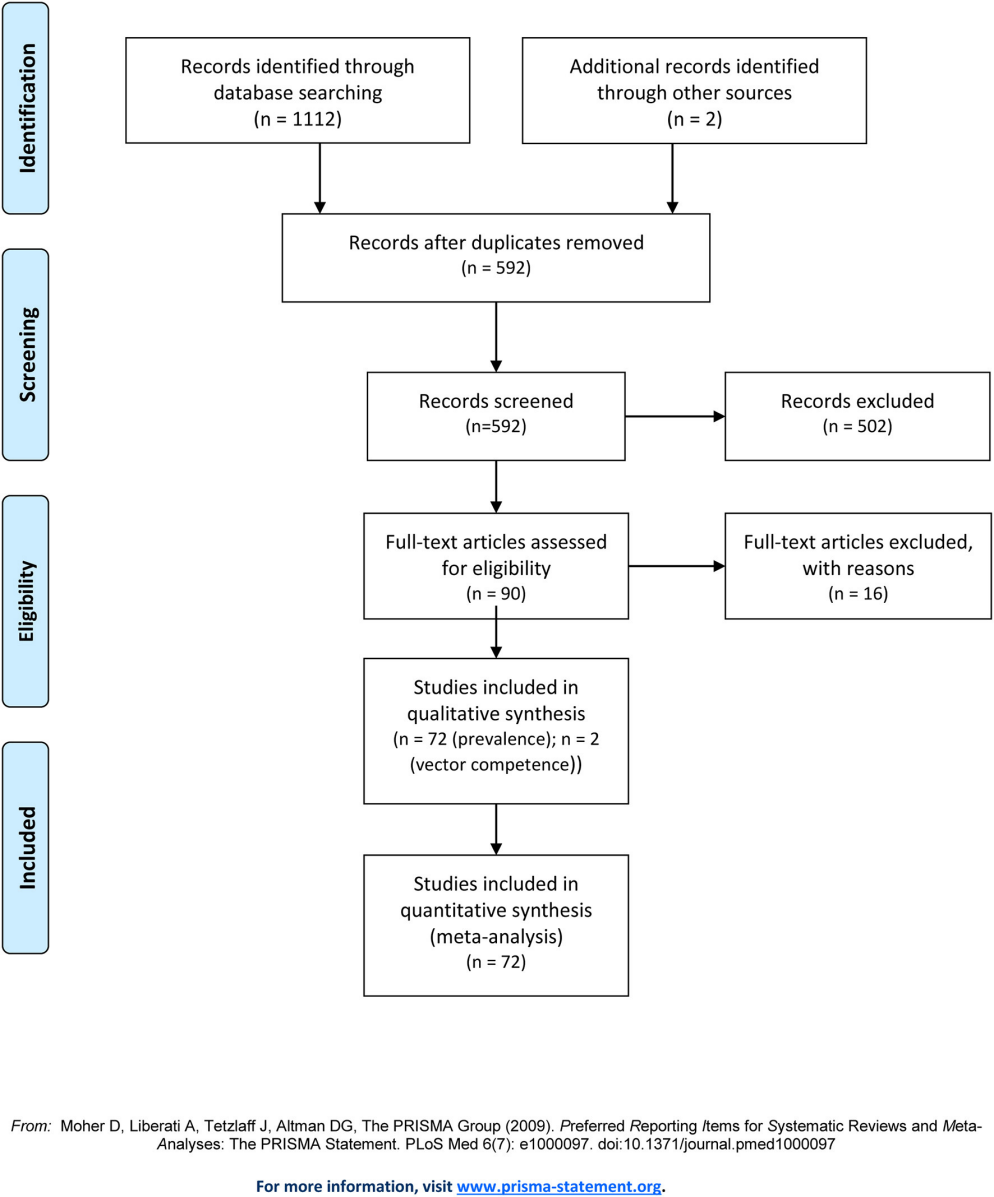
PRISMA flow chart for selection of studies, including the reasons for exclusion from the analysis.

A total of 590 studies were found using the mentioned search terms. Additionally, two studies using the platform Google Scholar were included. Based on the title and abstract, 502 studies were excluded. Main reasons for exclusion were non-European studies (*n* = 272), no hard ticks tested (*n* = 120), or reviews (*n* = 44); 66 studies were excluded for other reasons (Figure 1: PRISMA analysis). After full-text analysis, 16 further studies were removed. Finally, 72 publications in which the prevalence of *C. burnetii* in ticks collected in European countries were examined, compiled, and evaluated. Additionally, two vector competence studies were included.

### Prevalence Studies in Europe—A Systematic Analysis

Prevalence studies were analyzed by country, the number of tested ticks, tick species, the method used for tick collection, and the method used for detection of *C. burnetii* (Supplementary Table 1). The ticks tested in these studies were sampled between 1994 and 2018. In more than half of the studies (*n* = 44), ticks were collected between the years 2011 and 2013. Studies were performed in 23 of 45 different European countries.

Most collection areas were rural, single studies collected ticks in recreational areas in urban settings (72, 73). Furthermore, ticks were sampled from vegetation and animals on European islands (74–77).

In total, 115,265 ticks were tested in all 72 analyzed studies, of which 62,889 were sampled in a single and thus largest study performed in Switzerland (78). Excluding this study, on average, 689 ticks were sampled per study, ranging from 18 to 7,050 samples. The mean prevalence of *C. burnetii*-DNA over all evaluated studies was 4.8%. In half of the studies (*n* = 38), no *C. burnetii*-positive tick was identified. The highest prevalence was determined to be 54.2% in a Spanish study (79). Of the 34 studies with positive results, 10 (29.4%) confirmed the results with the sequencing of at least one PCR amplicon. Sequencing could not be performed in some cases because of low DNA yield (80). Ticks were sampled by flagging or dragging from the vegetation or removed from animals.

Most studies simultaneously gathered further information, e.g., about tick infestation of local animal species or other tick-borne pathogens. In addition, the seroprevalence for *C. burnetii* or other tick-borne agents of captive domestic and wild animals was examined (79–82).

#### Country

**Figure 2:**
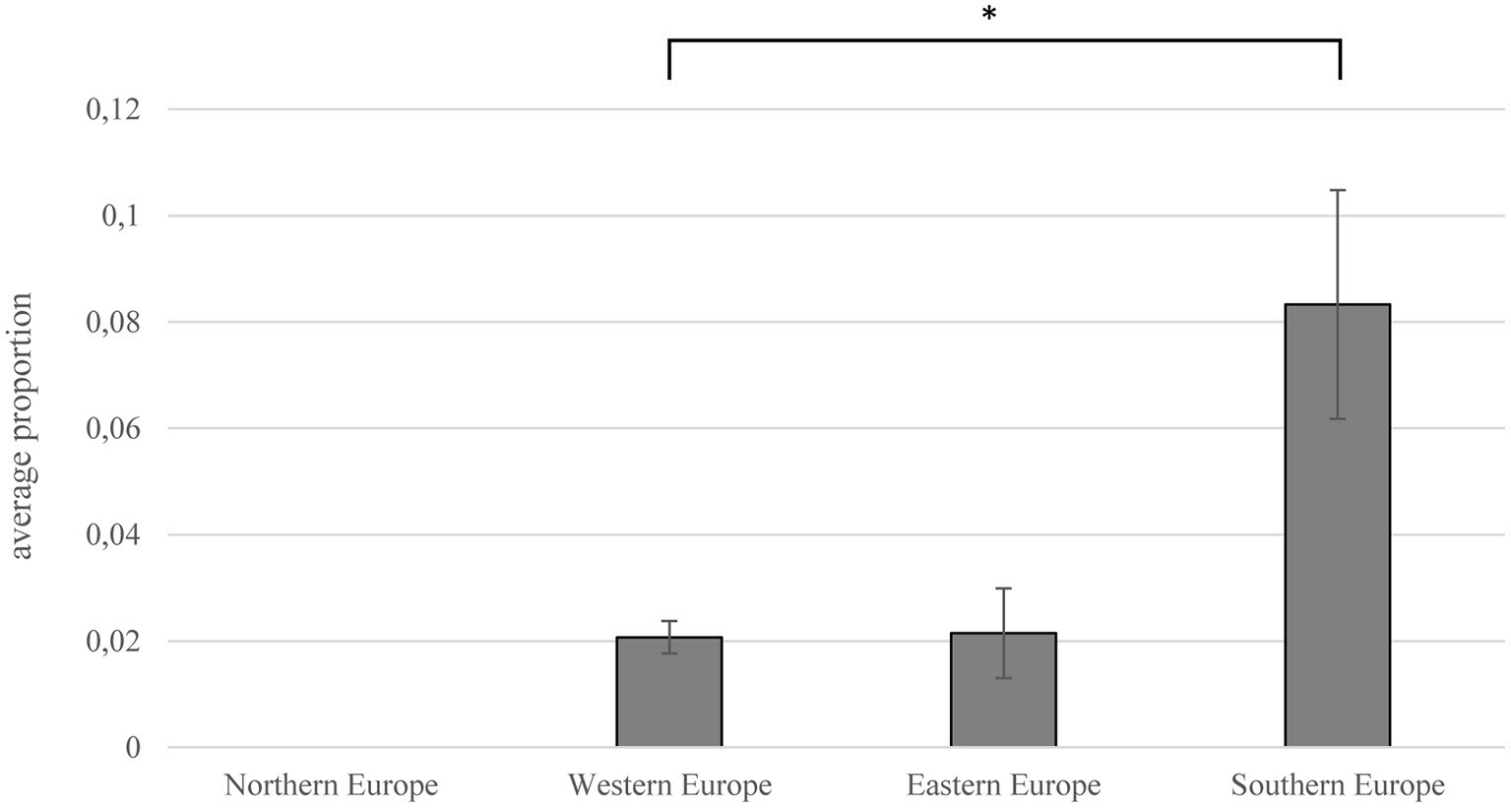
Prevalence (ratio of *C. burnetii*-positive ticks to total number of tested ticks) depending on European regions. Error bars show average confidence interval; significance was proven using Mann–Whitney U-test (*p = 0.013).

There were major differences in prevalence between different countries and regions in Europe (Figure 2, Supplementary Figure 1). The majority of studies were performed in Southern Europe (*n* = 30), whereas the number of prevalence studies per country was highest for Slovakia (*n* = 12). A high prevalence was found in studies in Southern Europe, especially in Spain (10.1%) and Portugal (10.6%), but also in Poland, a high proportion of ticks was positive (8.5%). In ticks tested in Northern Europe, no *C. burnetii* was detected (83–86).

The highest prevalence within single studies was determined in Spain and Italy. González et al. detected *Coxiella*-DNA in over 50% of the 236 tested ticks (79). In studies from Italy and France, more than 30% of tested ticks were found to be positive for *C. burnetii*-DNA (87–89). The mean prevalence in Southern Europe (8.3%) was significantly higher than in the rest of Europe (Mann–Whitney U test; *p* = 0.031), especially in Western Europe (2.1%) (Mann–Whitney U test; *p* = 0.013).

#### Tick Species

More than 25 tick species were examined in all studies (Table 1). In *Amblyomma* spp., *Haemaphysalis inermis, Hyalomma scupense, Hyalomma truncatum, Ixodes festai*, and *Ixodes hexagonus*, no molecular detection of *C. burnetii*-specific DNA was reported. Furthermore, in *Haemaphysalis hispanica, Hyalomma aegyptium, Hyalomma lusitanicum, Ixodes ventalloi, Rhipicephalus pusillus*, and *Rhipicephalus thuranicus, Coxiella*-DNA was detected, but the results were not sequenced for confirmation or discriminated from CLE.

**Table 1:** Selected tick species collected in Europe and detection of *C. burnetii* and CLE (Reference).

**Tick species**	**Molecular detection of *C. burnetii***	**samples sequenced**	**Vector competence studies**	**Detection of CLE**
*Dermacentor marginatus*	Yes (72, 90–97)	Yes (93)	Fecal excretion (98)	Yes (99)
*Dermacentor reticulatus*	Yes (82, 92, 100, 101)	Yes (82, 101, 102)	–	Yes (102, 103)
*Haemaphysalis concinna*	Yes (91, 101, 104, 105)	Yes (101)	–	–
*Haemaphysalis inermis*	No		–	Yes (102)
*Haemaphysalis punctata*	Yes (72, 80, 90, 93, 106)	Yes (93)	–	–
*Haemaphysalis sulcata*	Yes (74, 93, 95)	Yes (93)	–	–
*Hyalomma aegyptium*	Yes (107)	No	Transstadial transmission (108)	–
*Hyalomma lusitanicum*	Yes (79, 90, 94)	No		–
*Hyalomma marginatum*	Yes (76, 87, 88, 97)	Yes (76)	–	–
*Hyalomma rufipes*	Yes (88)	–	–	–
*Hyalomma scupense*	No	–	–	–
*Hyalomma truncatum*	No	–	–	–
*Ixodes acuminatus*	Yes (109)	Yes (109)	–	–
*Ixodes festai*	No	–	–	–
*Ixodes hexagonus*	No	–	–	Yes (99, 110)
*Ixodes ricinus*	Yes (72, 80, 81, 91, 92, 94, 96, 101, 102, 104, 105, 109, 111–117)	Yes (101, 102, 109, 112)	fecal excretion and transstadial transmission (98)	Yes (99, 102, 110, 118)
*Ixodes ventalloi*	Yes (96)	No	–	–
*Rhipicephalus annulatus*	Yes (76, 87)	Yes (76)		
*Rhipicephalus bursa*	Yes (76, 87, 93, 97)	Yes (76, 93, 97)	–	Yes (99, 119)
*Rhipicephalus pusillus*	Yes (90, 94)	No	–	–
*Rhipicephalus sanguineus*	Yes (74, 76, 90, 93, 96, 97, 120, 121)	Yes (76, 93, 95, 97)	historic: transstadial transmission (22)	Yes (99, 110, 122)
*Rhipicephalus turanicus*	Yes (72, 74)	No	–	Yes (122)

**Figure 3:**
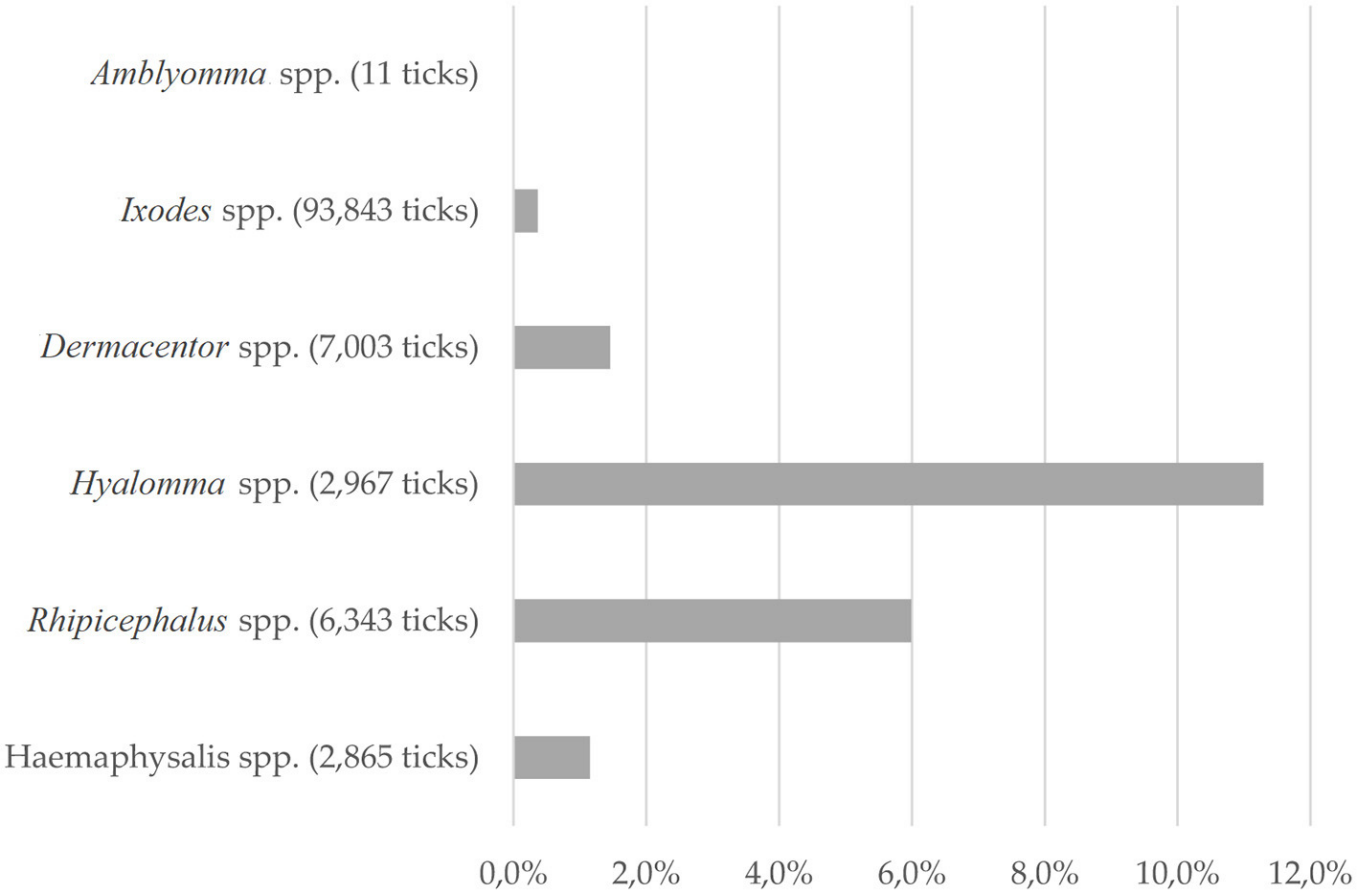
Tick species: Proportion of *C. burnetii*-positive ticks of the ticks tested, depending on tick genera. For 2,233 ticks, only total numbers were described.

Differences in detecting *C. burnetii*-positive ticks were noticed for the tick genera tested (Figure 3). The highest prevalence was observed in *Hyalomma* spp. ticks (11.3%). Of the six species tested, *Hyalomma rufipes, Hyalomma marginatum, H. lusitanicum*, and *H. aegyptium* were positive for *C. burnetii*. The most abundant and most often infected species was *H. lusitanicum*, in which 17.7% of ticks were PCR positive for *C. burnetii*. Furthermore, 6.0% of all tested *Rhipicephalus* spp. were positive for *C. burnetii*-DNA, whereas *Dermacentor* spp. harbored *Coxiella*-DNA in 1.4% of samples. By far, the most tested ticks belonged to the genus of *Ixodes* spp. (81.4%). Of these ticks, only 0.4% carried *C. burnetii*-DNA. The second least *Coxiella*-infested tick species was *Haemaphysalis* spp. with 1.2% positive samples. Of *Amblyomma* spp., only 11 negative ticks were flagged or removed from birds, which is not surprising, as this genus is not endemic in Europe (77, 90).

#### Collection Method

**Figure 4:**
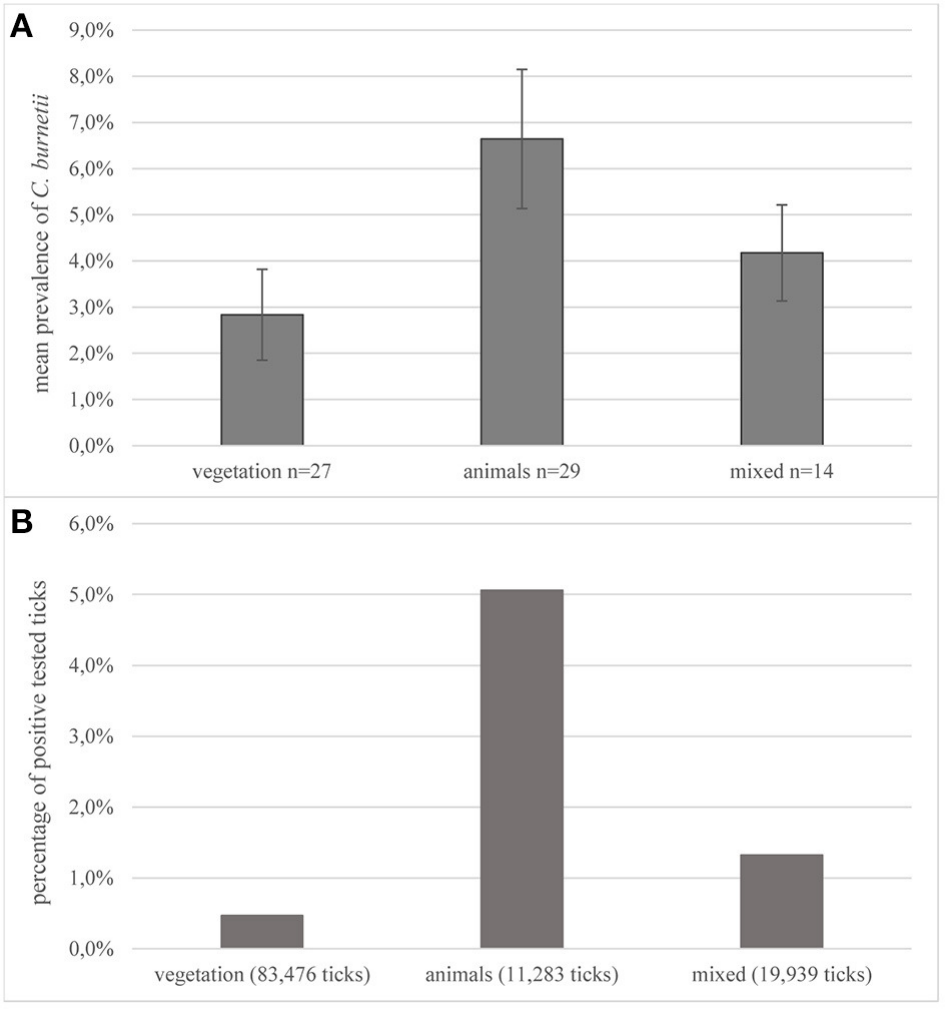
Collection methods. **(A)** Average prevalence of *C. burnetii* in studies on ticks depending on the collection method. Error bars show average confidence intervals. *n* = number of studies; **(B)** percentage of positively tested ticks of the total number of tested ticks in all studies, depending on the collection methods. Number in parentheses = total number of sampled ticks with this method.

The most common methods for prevalence studies were sampling from animals (*n* = 29, 40.3%; 11,283 ticks) or from the vegetation by dragging or flagging a piece of cotton (*n* = 27, 37.5%, 83,476 ticks). Overall, in the evaluated studies, 72.4% of all tested ticks were derived from vegetation. In 19.4% (*n* = 14; 19,939) of the examined studies, a combination of sampling from animals or humans and flagging was performed. Data in this review were not divided into the different collection methods used. Two studies examined the occurrence of pathogens in ticks exclusively collected from humans (123, 124). The highest mean prevalence for *C. burnetii* was found in studies in which ticks were removed from infected animals with 6.6%, whereas the mean prevalence in ticks collected from vegetation was 2.8% (Figure 4A). Of all tested ticks removed by flagging, 0.5% were positive for *C. burnetii*-DNA (Figure 4B). Tested animals ranged from pets and livestock to trapped rodents or game. In some studies, ticks were removed from birds to examine their role as hosts and vehicle for tick species (81, 88, 125).

*C. burnetii* was detected in two *H. marginatum*, feeding on humans in Sardinia. Contrary, in two studies that tested human-derived ticks exclusively, all were negative for *C. burnetii*-specific DNA (76, 123, 124).

The only study that was directly associated with a Q fever outbreak was performed in the Netherlands. Almost 3,000 ticks were sampled from nature, domestic animals, and wildlife. No *C. burnetii*-positive questing tick was found, whereas five female ticks collected from animals were positive for IS1111 and *com1* target sequences, suggesting uptake of blood from a bacteremic host (111). Further investigations revealed that these positive ticks were collected from recently vaccinated sheep. This might indicate a low risk of field infection during the outbreak.

#### Detection Method

**Figure 5:**
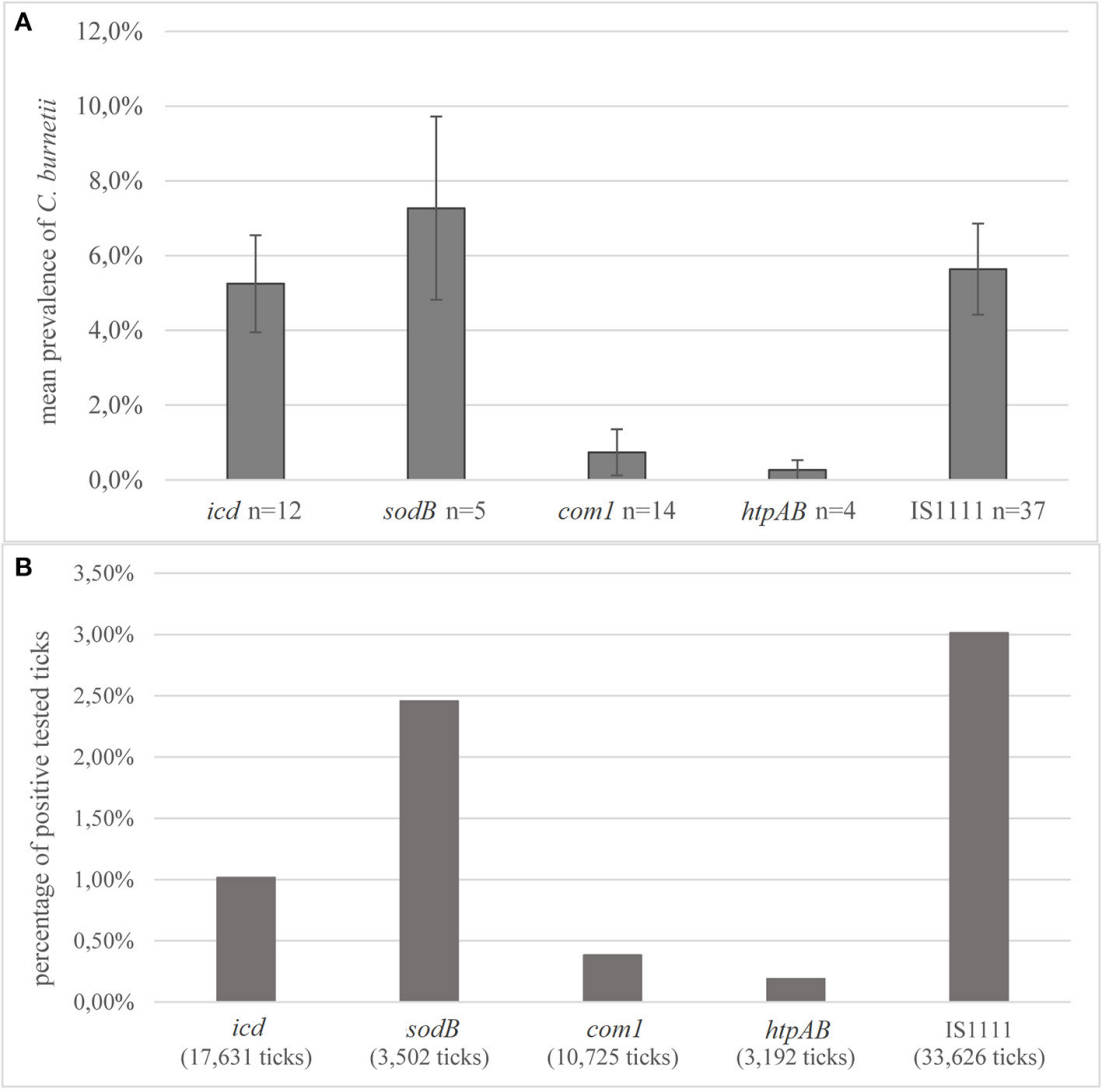
Detection methods **(A)** average prevalence of *C. burnetii* in PCR detection depending on the target genes. Error bars show average confidence intervals. *n* = number of studies; **(B)** percentage of positively tested ticks of the total number of tested ticks in all studies, depending on the detection method. In two studies, 567 ticks were collected from humans and tested negative. Number in parentheses = total number of ticks tested with this method.

Conventional PCR and real-time PCR were applied as a detection method in most of the studies. The predominant target genes for PCR detection were fragments of the insertion sequence IS1111 (*n* = 37, 51.4%, 33,626 ticks) or the genes *com1* (*n* = 14, 19.4%, 10,725 ticks), *icd* (*n* = 12, 16.7%, 17,631 ticks), *sodB* (*n* = 5, 6.9%, 3,502 ticks), and *htpAB* (*n* = 4, 5.6%, 3,192 ticks). Single studies used more than one target gene for identification (75, 85, 100, 126, 127). Of studies, which used *sodB* as the target gene for *C. burnetii*, an average prevalence of 7.3% was reported (Figure 5A). Of the studies using PCR targeting the *com1* fragment, the mean prevalence was 0.8%. The most frequently used target IS1111 resulted in an average prevalence of 5.7%. Of all ticks tested with IS1111, 3.0% were positive, which is the highest percentage (Figure 5B). The largest sampling size of 62,889 ticks, exclusively *I. ricinus*, was collected in a study in Switzerland. No *C. burnetii*-positive tick was detected by using *ompA* as the target gene in this study (78). Also, 16S ribosomal RNA (rRNA) sequencing was used for the detection of *Coxiella* spp. in eight studies; one of them detected *C. burnetii*-positive ticks (102).

Before PCR detection, some studies used the hemocyte test for visual detection of *Coxiella* spp. and morphologically similar agents in the hemolymph (91, 104). In these studies, *C. burnetii* was detected by PCR in four hemocyte-positive ticks, but also in two hemocyte-negative ticks.

Furthermore, studies used non-molecular methods in addition to PCR, such as cultivation in cells or embryonated eggs, resulting in the isolation of *Spiroplasma* spp. but not *C. burnetii* (126).

#### Coxiella-Like Endosymbionts

Some authors considered CLE and searched specifically for these endosymbionts or detected CLE by sequencing positive PCR products. Furthermore, sequencing of 16S rRNA was used to evaluate the tick microbiome (99, 103). In seven studies, CLEs were detected additionally or exclusively using sequencing in a variety of tick species, or *Coxiella* was only identified on genus level (99, 102, 103, 110, 119, 122). Nine tick species were found to harbor CLE in these studies.

### Experimental Vector Competence Studies

In the decades after the first isolation of *C. burnetii* from a tick, experimental transmission studies were conducted on various tick species. However, only two recent investigations on the vector competence of ticks under laboratory conditions were found in the literature.

In one study, the vector competence of the tick *H. aegyptium* was proven, including the transstadial transmission over all life stages (108). Larvae were fed on infected guinea pigs, and 5.6% tested positive after molting into nymphs. After feeding on an infected host, 28.9% of nymphs molted into adults remained positive. In addition, reinfection of uninfected guinea pigs was shown in this study.

For *I. ricinus*, a transstadial transmission from nymphs to adults was shown using an *in vitro* feeding system, enabling better differentiation between the transmission *via* saliva or feces (98). The transstadial transmission rate from nymphs to adults was determined to be 25%, but horizontal transfer into the blood was not shown, whereas the ticks excreted infectious feces during feeding and after molting.

## Discussion

### Higher Prevalence in the Mediterranean Region Might Be Associated With Regional Distribution of Different Tick Species

Duron et al. found an average prevalence of 5% of *C. burnetii* in ticks after evaluation of 60 studies conducted worldwide (28). The data of the present study show a similar mean prevalence of studies on ticks in Europe. Here, an update is provided, enhancing the insight into the prevalence of *C. burnetii* in various tick species, demonstrating that there is a huge variation between the results of different studies depending on the country, tested species, method of collection, and *C. burnetii* detection method applied.

Evaluating the studies, a gradient from North to South was noticed, resulting in a higher prevalence in the Mediterranean region, followed by Eastern Europe. Most positive samples were obtained in Southern Europe, especially Portugal, Spain, and Greece. Here, species of the genera *Hyalomma, Rhipicephalu*s, and *Haemaphysalis* are the most abundant vector ticks. Due to climate change associated with global warming, the spread of arthropods is expected (128), which could possibly lead to an increased role of pathogen transmission by these tick species in Western and Northern Europe. The tick species *H. marginatum* was detected in the last years more often in Central Europe and hibernation of this species was recently observed in Germany (64). Since in most of the studies, no confirmation of positive results by specific methods for *C. burnetii* was performed, an inadvertent detection of CLE cannot be excluded. However, this differentiation is extremely important because CLEs seem to have less if at all pathogenic potential than *C. burnetii*.

*Coxiella burnetii* is rarely found in ticks in most of the regions in Europe. It is presumed that ticks play a minor role in Q fever transmission, also considering that no validated human cases of *C. burnetii* infection via ticks have been reported. Foci in which ticks may play a role as the natural reservoir for Q fever seem to exist, but these hot spots are hard to detect. Similar patterns of regional foci were described for tick-borne encephalitis virus (129, 130). The absence of *C. burnetii*-infected ticks in endemic areas is often explained by narrow hot spots (131). That causes less frequent surveillance of this pathogen in major prevalence studies, mainly focusing on well-described tick-borne infectious agents such as *Anaplasma phagocytophilum* or *B. burgdorferi* s.l. Under these circumstances, there is a lack of data in certain countries and regions. The evaluation of European studies shows that occurrence of *C. burnetii* is highly dependent on the tick species. The most abundant tick species in Europe, *I. ricinus*, seems to be infected with *C. burnetii* very rarely. Occasional reports of successful isolation of *C. burnetii* from field-collected *I. ricinus* support the theory of less relevance in Q fever transmission (132). In the past, a correlation was presumed between the occurrence of Q fever and the abundance of *Dermacentor marginatus* (133). Considering the low prevalence in this species, this theory was not proven by the data collected here from the analyzed studies. However, there seems to be an association of *C. burnetii* with ticks of the genus *Hyalomma* spp. and *Rhipicephalus* spp., naturally present in regions with ambient temperatures, e.g., the Mediterranean region. This is also supported by the *in vivo* experimental study performed with *H. aegyptium* (108). As *Rhipicephalus* spp. is highly infected with CLE, positive results should be confirmed by sequencing (41, 119).

Prevalence studies targeting *C. burnetii* in ticks are conducted worldwide, and positive ticks were found on all continents except Antarctica (134–138). Comparably with European studies, there are divergent findings in the different investigations. Recently, a study conducted in China using 16S rRNA sequencing reported a prevalence of 40–96% depending on tested species (139). High prevalence was also found in several countries, e.g., Argentina, Egypt, or Nigeria (140–142). Apart from that, in studies in Japan or Reunion Island, no *Coxiella*-positive ticks were determined (143, 144). Despite the origin of the first isolate, *C. burnetii* is reported rarely from ticks in North America (145, 146).

### A Combination of Different Collection Methods Supports a Realistic Depiction of Reality

Different collection methods were used in the studies, but the most common is removing from animals or flagging. Simple detection of the DNA of a pathogen does not prove the vector competence of a certain tick species (92, 147). Ticks can also carry a pathogen without the ability to transmit it (93). These arthropods feed on bacteraemic/viraemic/protozooaemic animals and are likely to take up any agent circulating in the blood of the host. The slightly higher prevalence of *C. burnetii* in ticks collected from animals compared with ticks from vegetation argues in favor of such an uptake. The procedure of flagging of questing ticks, therefore, seems to be more appropriate for information regarding the prevalence and to discriminate between the role of ticks as a reservoir or as an accidental host (148). Additionally, data about the local seroprevalence of *C. burnetii* in animal host species could be included. A combination of both origins, vegetation, and animals, might also be of interest, especially in the context of outbreak surveillance. Moreover, only exophilic and questing ticks can be obtained using the dragging method. That excludes, for example, premature life stages of *Dermacentor* spp. or preferably hunting ticks, e.g., *Hyalomma* spp. As it is known that *C. burnetii* genomes found in ticks genetically cluster with samples isolated from wildlife, the inclusion of hunted animals can increase the knowledge of potentially sylvatic cycles (149). The sampling of ticks from migratory birds can give insight into the movement of ticks and the pathogens or microbial communities they carry. Examination of avian ticks and tick-borne diseases can also help monitor the spread of these ticks and their pathogenic cargo (150). The introduced tick species might not be adapted to the climate and availability of host species in this country; therefore, its survival is unclear. Sampling on farm animals and game favors a higher rate of adult ticks, as this life stage is more frequently feeding on larger animals (112). Examination of ticks removed from human patients also might be of interest regarding the zoonotic potential of the disease. In a study by Dubourg et al., patients showing scalp eschar were examined, and removed ticks were tested for a wide range of tick-borne pathogens. Of the 11 ticks, mainly *D. marginatus*, two carried *C. burnetii*, whereas *Rickettsia slovaca* was the most prevalent pathogen (151). Simultaneous infection of human patients with *C. burnetii* and other tick-borne pathogens were described, but it cannot be excluded that the temporal connection between the infections is random, as Q fever is endemic worldwide, and infection might have been caused by inhalation (30). To determine the significance of ticks in Q fever transmission, it might be of major interest to perform prevalence studies in areas of active Q fever outbreaks or in known *Coxiella*-positive herds. This is important to prove any association between infected animals or humans and the local arthropods. Furthermore, identification of tick life stage is important to analyze the possibility of transovarial transmission. *Coxiella burnetii* is considered one of the most relevant pathogens and should be prioritized in the examination of wildlife (152).

### Prevalence Studies Should Include Specific *C. burnetii* Detection Methods

There was no significant difference noticed between the various target genes used in the evaluated studies. The determined prevalence depends, among other things, on the sensitivity of the used target gene. Some assays, especially when using IS1111, detect lower amounts of DNA and thus are more sensitive than others. This relation can be described more precisely by the use of standardized controls such as plasmids. Because of the close genetic relationship to CLE, the high specificity of molecular methods is required. Because frequently used PCR target gene sequences, e.g., IS1111, were also detected in endosymbionts, there is a need for specific methods to distinguish between these species. This would rule out an overestimation of the dissemination of *C. burnetii* within the tick population. The majority of studies detecting commonly used target genes for the detection of *C. burnetii* did not confirm the results with sequencing. Particularly, the IS1111 fragment is a common target for *C. burnetii* detection in ticks, which is known to be less specific but highly sensitive for the pathogen (57). The specificity for detection of *C. burnetii* in tick samples is limited in all commonly used target genes; thus, the use of single targets is not recommended (56, 58). Positive results in PCR should be interpreted carefully, and sequence confirmation should be mandatory, preferably using long targets for increased specificity (56, 153). Due to low DNA yield, sequencing might not be possible. In these cases, the combination of different target genes for detection could be a valid method to minimize the risk of unspecific results. Thus, there is a need to develop specific assays for differentiation between *C. burnetii* and CLE, which might also be applicable for higher sample sizes and poor DNA yield.

### Unclear Pathogenic Potential of *Coxiella*-Like Endosymbionts Should Not Be Neglected

In seven studies, CLEs were detected in tick samples, mainly using sequencing of 16s rRNA. These endosymbionts are distributed in several hard and soft tick species and represent a large proportion of the microbiome of some species (33, 154). This leads to the presumption that the specificity of some molecular methods may not be sufficient to distinguish between *C. burnetii* and endosymbionts. Several *Amblyomma* spp. and *Rhipicephalus* spp. were shown to be CLE carriers in up to 100% of analyzed tick samples (41). In another study with more than 50 different tick species of hard and soft ticks, more than two-thirds of the species were found to harbor CLE (38). *Coxiella*-like endosymbionts seem to be associated with some genera, for example, *Rhipicephalus* spp. or *Ornithodoros* spp., in which CLEs were detected with a high prevalence, whereas only a few positive samples originated from *Ixodes* spp. Binetruy et al. found CLE to be present in 11 of 24 species of the genus *Amblyomma* (155). Recent phylogenetic analysis revealed a close and apparently ancient alliance between *Rhipicephalus* spp. and their CLE (156). The main survival strategy of CLE is the vertical transmission *via* the egg, but also horizontal transfer, possibly *via* co-feeding, was proven (156, 157). Recent results based on genome sequencing have shown that certain CLEs seem to have evolved from an ancestor capable of infection of immune cells (158). Hence, a loss of pathogenic potential was suggested. In contrast, *C. burnetii* was reported to have its phylogenetic origin in CLE (38).

Novel tick-borne pathogens are emerging, and as the evolution of *C. burnetii* is closely linked to endosymbionts, the possibility of other *Coxiella* spp. being pathogenic should not be neglected, considering that bird infections or human skin infections were reported (38, 49, 103, 158). Increasing the knowledge on evolutionary processes and the pathogenic potential of CLE could likewise also contribute to a better understanding of the epidemiology of *C. burnetii*.

Little is known about the impact CLE might have on pathogen transmission. A reduced infection rate of *R. haemaphysaloides* with CLE correlated with a lower rate of transstadial transmission of *Babesia microti. Candidatus Midichloria mitochondrii*, another tick endosymbiont, is known to influence the occurrence and ability to detect tick-borne pathogens in *I. ricinus* (159, 160).

### Experimental Studies Are Needed to Assess the Vector Competence of Further Tick Species

In the past, seven tick species were shown to be competent vectors for *C. burnetii* (28). There is little recent research exploring the vector competence of ticks under laboratory conditions. Experimental studies prove transstadial transmission and successful reinfection of guinea pigs by *H. aegyptium* ticks (108) and transstadial transmission from nymphs to adults in *I. ricinus* (98). Recently, a transstadial transmission from nymphs to adults and a subsequent excretion with saliva were concluded in naturally infected *H. lusitanicum* (161). There is a lack of vector competence studies on different tick species, focusing on the transstadial and transovarial transmission of pathogens. However, those studies are limited by the low rate of transmission and consequently incomplete knowledge of epidemiological cycles (162). Furthermore, studies are missing, which describe the level and duration of bacteremia in *C. burnetii*-infected hosts, which are necessary to estimate the actual vector capacity under laboratory conditions. As a mainly airborne pathogen, the potential infection routes may be *via* inhalation of feces besides the injection of saliva during the tick bite (98, 108). Infected feces might also contaminate the wound and thus causing an infection, as it is known from the transmission of *Trypanosoma cruzi* by bed bugs or *Rickettsia prowazekii* and lice (163, 164).

Besides the need for information regarding the prevalence of *C. burnetii* in ticks, the risk of acquiring Q fever by a tick bite cannot finally be determined. In different studies, the correlation between coxiellosis and the abundance of ticks as a risk factor was examined. Predominantly, no significant correlation was found (165–167), whereas other investigations concluded an association between tick infestation and seroprevalence (168, 169).

Most tick species in Europe are spreading, and the increased risk of tick-borne diseases accompanies this. For this reason, extensive and focused monitoring of ticks and their microbial burden is crucial, as well as further research on possibly tick-borne diseases and the pathogenic potential of already known bacteria.

## Conclusions

The evaluation of European studies shows a significantly higher prevalence of *Coxiella* spp. in ticks in Mediterranean countries. This is likely to be driven by the abundance of different tick species in these countries, i.e., *Hyalomma* spp. and *Rhipicephalus* spp. In the context of global warming, the geographical distribution of these tick species changes and thus the epidemiology of Q fever in currently temperate Europe. Based on the large number of studies, which failed to detect *C. burnetii* DNA in tick samples, ticks carrying *C. burnetii* seem to be restricted to certain regions. Positive results have to be interpreted carefully because no distinction between *C. burnetii* and CLE was made in most of these studies. Planning of a prevalence study on ticks should, therefore, in particular, focus on the choice of detection methods for specific results. Methods should aim to differentiate *C. burnetii* and CLE using sequencing or at least to use different target genes in positive samples.

To address the questions on the role ticks play as a reservoir in Q fever transmission, vector competence studies using different and relevant tick species should be performed.

## Data Availability Statement

The original contributions presented in the study are included in the article/Supplementary Material, further inquiries can be directed to the corresponding author/s.

## Author Contributions

SK and KM-S designed the study, SK conducted the literature search, SK, GM, SU, MP, and KM-S performed the data extraction, and SK analyzed the data and drafted the manuscript. All authors contributed to the article and approved the submitted version.

## Conflict of Interest

The authors declare that the research was conducted in the absence of any commercial or financial relationships that could be construed as a potential conflict of interest.

## Supplementary Material

Supplementary Figure 1 Forest plots of the included prevalence studies.

Supplementary Table 1 Overview on included prevalence studies and the collected data.
