# Supplementary material for: The Prevalence of Coxiella burnetii in Hard Ticks in Europe and Their Role in Q Fever Transmission Revisited—A Systematic Review
Source: Front Vet Sci. 2021 Apr 26;8:655715. doi: 10.3389/fvets.2021.655715 (PMC8109271; doi:10.3389/fvets.2021.655715)
Supplement: Supplementary Figure 1 — Forest plots of the included prevalence studies. [file Data_Sheet_1.PDF]

Figure S1: Forest plot showing the prevalence or MIR and CI, sorted by the four different European regions.

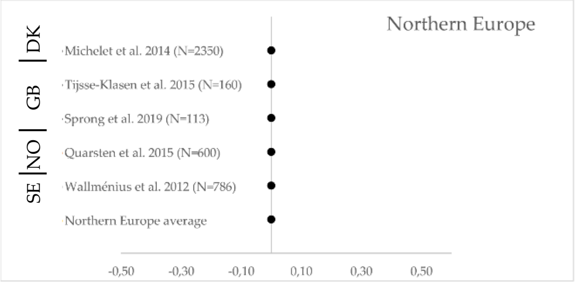

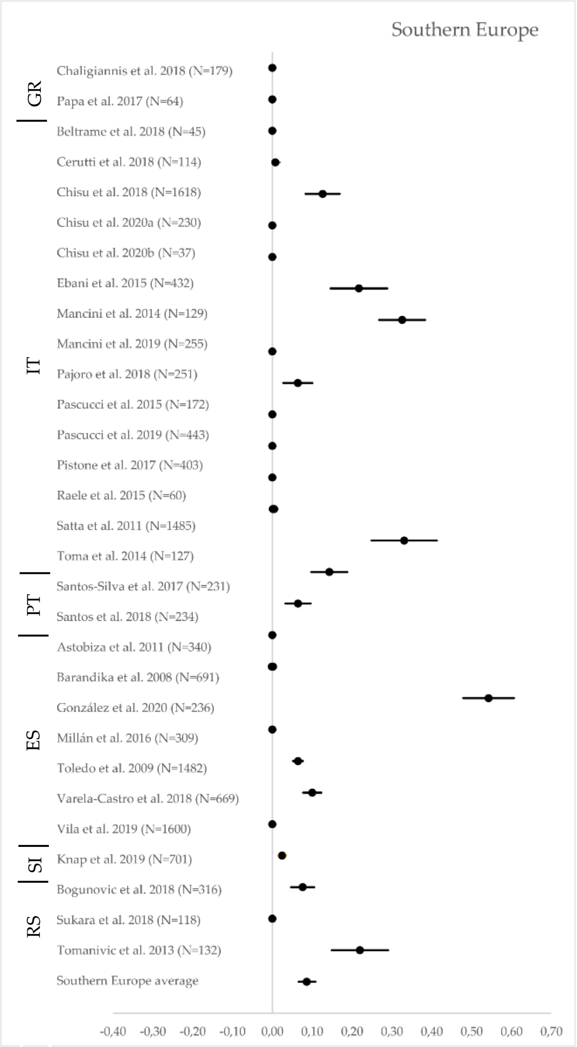

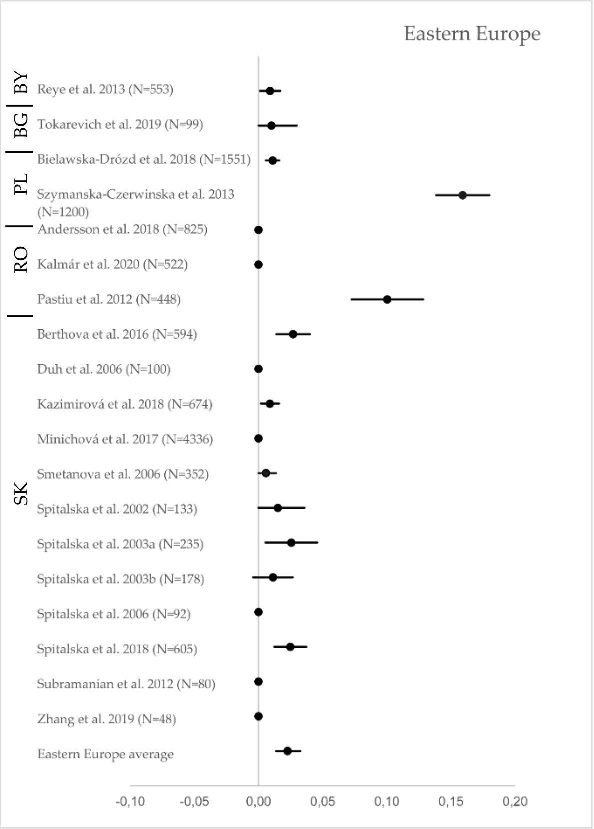

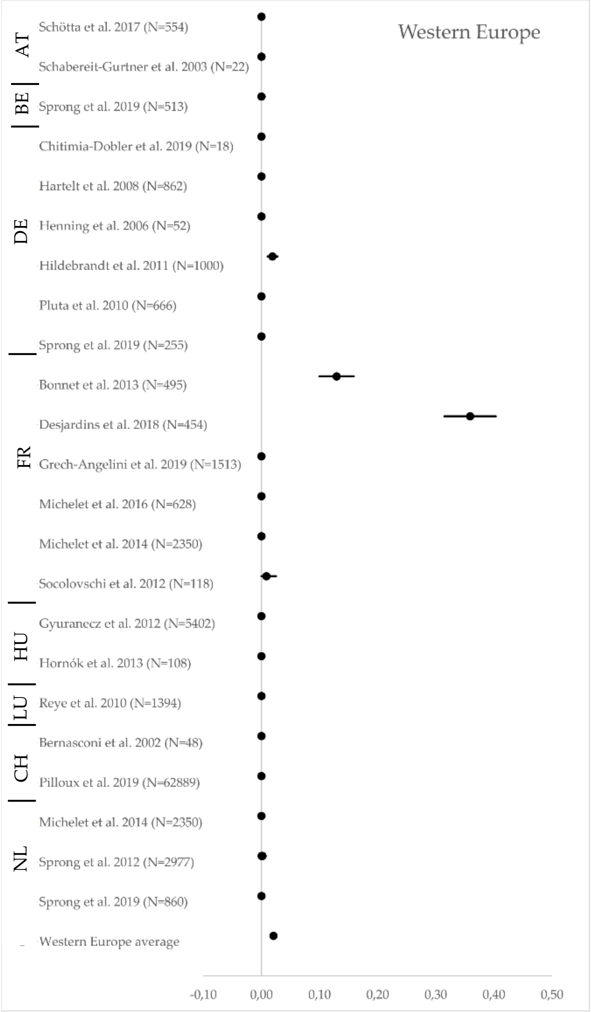

Countries are abbreviated according ISO 3166-1.
DK Denmark; GB Great Britain; NO Norway; SE Sweden; GR Greece; IT Italy; PT Portugal; ES Spain; SI Slovenia; RS Serbia; BY Belarus; BG Bulgaria; PL Poland; RO Romania; SK Slovakia; AT Austria; BE Belgium; DE Germany; FR France; HU Hungary; LU Luxembourg; CH Switzerland; NL The Netherlands
