# Supplementary material for: The Prevalence of Coxiella burnetii in Hard Ticks in Europe and Their Role in Q Fever Transmission Revisited—A Systematic Review
Source: Front Vet Sci. 2021 Apr 26;8:655715. doi: 10.3389/fvets.2021.655715 (PMC8109271; doi:10.3389/fvets.2021.655715)
Supplement: Supplementary Table 1 — Overview on included prevalence studies and the collected data. [file Table_1.DOCX]

**Table S1.** Included studies on prevalence of C. burnetii in ticks in Europe

|  | **Reference** | **Country** | **Tick species** | **N** | **n** | **Proportion (CI)** | **Method/Target gene** | **origin** | **Sequenced** | **CLE** |
| --- | --- | --- | --- | --- | --- | --- | --- | --- | --- | --- |
| (1) | Andersson et al. 2018 | Romania | D.m. (0/92), D.r. (0/119), H.c. (0/3), H.p. (0/38), Hy.m. (0/26), Hy.s. (0/1) I.c. (0/7), I.r. (0/209), R.b. (0/15), R.r. (0/1), R.s. (0/314) | 825^2^ | 0 | 0.00^1^ | qPCR (com1, icd, IS1111) | mixed |  |  |
| (2) | Astobiza et al. 2011 | Spain | D.m., D.r., H.p., I.h., I.r. | 340 | 0 | 0.00 | qPCR (IS1111) | animals |  |  |
| (3) | Barandika et al. 2008 | Spain | D.r. (0/97), H.c. (0/52), H.i. (0/95), H.p. (1/109), I.r. (0/288), R.b. (0/50) | 691 | 1 | 0.00 | PCR, Hybridization (*htpAB*) | vegetation | no |  |
| (4) | Beltrame et al. 2018 | Italy | I.r. (0/43), R.s. (0/2) | 45 | 0 | 0.00 | qPCR (IS30a) | humans |  |  |
| (5) | Bernasconi et al. 2002 | Switzerland | D.m. (0/1), H.spp. (0/1), R.s. (0/24), R.t. (0/22) | 48 | 0 | 0.00 | PCR (16S rDNA) | animals |  | R.s., R.t. |
| (6) | Berthova et al. 2016 | Slovakia | I.r. (16/594) | 594 | 16 | 0.03 (0.01-0.04) | PCR (*com1*) | animals | no |  |
| (7) | Bielawska-Drózd et al. 2018 | Poland | I.r. (17/1551) | 1551 | 17 | 0.01 (0.01-0.02) ^1^ | qPCR (IS1111) | vegetation | no |  |
| (8) | Bogunovic et al. 2018 | Serbia | D.r. (0/1), I.r. (0/87), R.s. (24/228) | 316 | 24 | 0.08 (0.05-0.11) | PCR (IS1111) | animals | yes |  |
| (9) | Bonnet et al. 2013 | France | D.m. (44/377), D.r. (12/74), I.r. (8/44) | 495 | 64 | 0.13 (0.10-0.16) | qPCR (IS1111) | vegetation | no |  |
| (10) | Cerutti et al. 2018 | Italy | I.r., Hy.spp., Hy.d./m., Hy.l., A.spp., I.ar. | 114 | 0 | 0.00 | qPCR (IS1111) | animals |  |  |
| (11) | Chaligiannis et al. 2018 | Greece | D.m. (pos), H.pa. (pos), H.p., H.s. (pos), I.g. (pos), R.b., R.s. (pos) | 179 | 32 | 0.18 (0.12-0.23) | qPCR (IS1111) | animals | yes  (7 samples) |  |
| (12) | Chisu et al. 2018 | Italy | D.m. (0/272), H.p. (0/71), H.s. (0/30), Hy.l. (0/73), Hy.m. (2/45), I.fe. (0/6), R.a. (2/11), R.b. (3/400), R.s. (14/710) | 1618 | 21 | 0.01 (0.01-0.02) | PCR (*sodB*) | mixed | yes |  |
| (13) | Chisu et al. 2020a | Italy | D.m. (1/15), H.p. (0/5), H.s. (0/9), Hy.l. (0/1), Hy.m. (1/8), I.spp. (0/1), I.fe. (0/1), R.spp. (0/7), R.b. (5/34), R.s. (22/149) | 230 | 29 | 0.13 (0.08-0.17) | PCR (*sodB*) | animals | yes |  |
| (14) | Chisu et al 2020b | Italy | I.r. (0/37) | 37 | 0 | 0.00 | PCR (*sodB*) | animals |  |  |
| (15) | Chitimia-Dobler et al. 2019 | Germany | Hy.m. (0/10), Hy.r. (0/8) | 18 | 0 | 0.00 | qPCR (IS1111) | mixed |  |  |
| (16) | Desjardins et al. 2018 | France | D.spp. (2/5), H.spp. (5/21), Hy.spp. (16/45), R.spp. (140/383) | 454 | 163 | 0.36 (0.31-0.40) | qPCR (IS1111) | animals | no |  |
| (17) | Duh et al. 2006 | Slovakia | D.r. (0/100) | 100 | 0 | 0.00 | PCR(IS1111) | vegetation |  |  |
| (18) | Ebani et al. 2015 | Italy | D.m. (0/72), H.p. (0/30), I.r. (0/330) | 432 | 0 | 0.00^1^ | PCR (IS1111) | animals |  |  |
| (19) | González et al. 2020 | Spain | Hy.l. (128/236) | 236 | 128 | 0.54 (0.48-0.61) | qPCR (IS1111) | animals | no |  |
| (20) | Grech-Angelini et al. 2019 | France | D.m. (0/156), H.p. (0/74), H.s. (0/2), Hy.m. (0/362), Hy.s. (0/135), I.r. (0/115), R.a. (0/11), R.b. (0/508), R.s. (0/150) | 1513 | 0 | 0.00 | qPCR (*icd*, IS1111) | animals |  |  |
| (21) | Gyuranecz et al. 2012 | Hungary | D.spp. (0/4), D.m. (0/369), D.r. (0/361), H.c. (0/735), H.i. (0/315), H.p. (0/22), I.ac. (0/374), I.r. (0/3222) | 5402 | 0 | 0.00 | qPCR (IS1111) | mixed |  |  |
| (22) | Hartelt et al. 2008 | Germany | D. spp. (0/862) | 862 | 0 | 0.00 | nested PCR (IS1111) | vegetation |  |  |
| (23) | Henning et al. 2006 | Germany | I.spp. (0/52) | 52 | 0 | 0.00 | PCR (*com1*, *mucZ*) | animals |  |  |
| (24) | Hildebrandt et al. 2011 | Germany | I.r. (19/1000) | 1000 | 19 | 0.02 (0.01-0.03) | qPCR (IS1111, *icd*) | vegetation | no |  |
| (25) | Hornók et al. 2013 | Hungary | H.c. (0/1), Hy.m. (0/3), I.r. (0/104) | 108 | 0 | 0.00 | qPCR(IS1111) | animals |  |  |
| (26) | Kalmár et al. 2020 | Romania | I.r. (0/522) | 522 | 0 | 0.00 | qPCR (IS1111) | humans |  |  |
| (27) | Kazimirová et al. 2018 | Slovakia | H.c. (0/33), I.r. (6/641) | 674 | 6 | 0.01 (0.00-0.02)^1^ | PCR (*com1*) | animals | no |  |
| (28) | Knap et al. 2019 | Slovenia | D.r. (0/10), H.p. (1/65), I.r. (6/626) | 701 | 17 | 0.02 (0.01-0.04) | qPCR (IS1111) | mixed | no |  |
| (29) | Mancini et al. 2014 | Italy | D.m. (1/7), H.p. (1/4), I.r. (3/33), R.t. (23/85) | 129 | 28 | 0.22 (0.15-0.29) | qPCR (*icd*) | vegetation | no |  |
| (30) | Mancini et al. 2019 | Italy | Hy.m. (4/12), R.a. (70/215), R.b. (9/28) | 255 | 83 | 0.33 (0.27-0.38) | qPCR (*icd*; IS1111) | vegetation | no |  |
| (31) | Michelet et al. 2014 | France | I.r. (0/2350) | 2350 | 0 | 0.00 | qPCR (*icd*; IS1111) | vegetation |  |  |
|  |  | Denmark | I.r. (0/2350) | 2350 | 0 | 0.00 | qPCR (*icd*; IS1111) | vegetation |  |  |
|  |  | The Netherlands | I.r. (0/2350) | 2350 | 0 | 0.00 | qPCR (*icd*; IS1111) | vegetation |  |  |
| (32) | Michelet et al. 2016 | France | D.m. (0/67), D.r. (0/29), H.p. (0/532) | 628 | 0 | 0.00 | qPCR (*icd*) | vegetation |  |  |
| (33) | Millán et al. 2016 | Spain | I. spp. (0/43), R.s. (0/266) | 309 | 0 | 0.00 | qPCR (16S rRNA) | animals |  |  |
| (34) | Minichová et al. 2017 | Slovakia | D.m. (0/182), H.c. (0/166), H.c. (0/5), I.r. (0/3983) | 4336 | 0 | 0.00 | qPCR (*com1*) | mixed |  |  |
| (35) | Pajoro et al. 2018 | Italy | I.r. (0/251) | 251 | 0 | 0.00 | PCR (IS1111) | animals |  |  |
| (36) | Papa et al. 2017 | Greece | D.m. (0/11), H.pa. (0/2), I.r. (0/10), R.spp. (0/1), R.b. (0/37), R.s. (0/3) | 64 | 0 | 0.00^1^ | PCR (16S rRNA) | animals |  | D.m., I.r, H.pa., R.s., R.b. |
| (37) | Pascucci et al. 2015 | Italy | D.m. (0/1), H.p. (0/2), Hy.spp. (0/1), I.spp (1/7), I.ac. (6/92) I.h. (0/2), I.r. (4/23), R.t. (0/44) | 172 | 11 | 0.06 (0.03-0.10) ^1^ | qPCR (IS1111) | animals | yes |  |
| (38) | Pascucci et al. 2019 | Italy | A.spp (0/4), A.v. (0/1), Hy.spp. (0/54), Hy.m. (0/10), Hy.r. (0/366), Hy.t. (0/3), I.fr. (0/2), I.r. (0/3) | 443 | 0 | 0.00 | qPCR (IS1111) | animals |  |  |
| (39) | Pastiu et al. 2012 | Romania | Hy.ae. (45/448) | 448 | 45 | 0.10 (0.07-0.13) | PCR (IS1111) | animals | no |  |
| (40) | Pilloux et al. 2019 | Switzerland | I.r. (0/62889) | 62889 | 0 | 0.00^1^ | qPCR (*ompA*) | vegetation |  |  |
| (41) | Pistone et al. 2017 | Italy | I.r. (0/403) | 403 | 0 | 0.00 | PCR (IS1111) | mixed |  |  |
| (42) | Pluta et al, 2010 | Germany | D.m. (0/77), D.r. (0/133), D.spp. (0/456) | 666 | 0 | 0.00 | nested PCR (IS1111) | vegetation |  |  |
| (43) | Quarsten et al. 2015 | Norway | I.r. (0/600) | 600 | 0 | 0.00^1^ | qPCR (*icd*) | vegetation |  |  |
| (44) | Raele et al. 2015 | Italy | R.b. (0/60) + 40 egg samples | 60 | 0 | 0.00 | PCR (IS1111), LAMP (*com1*) | animals |  | R.b. |
| (45) | Reye et al. 2010 | Luxembourg | I.r. (0/1394) | 1394 | 0 | 0.00 | nested PCR (htpB) | vegetation |  |  |
| (46) | Reye et al. 2013 | Belarus | D.r. (0/226), I.r. (5/327) | 553 | 5 | 0.01 (0.00-0.02) | nested PCR (htpB) | mixed | yes |  |
| (47) | Santos et al. 2018 | Portugal | D.m. (1/2), H.i. (0/2), H.p. (0/13), I.f. (2/12), I.r. (1/37), I.v. (9/92), R.p. (0/22), R.s. (2/54) | 234 | 15 | 0.06 (0.03-0.10) | qPCR (IS1111); nested PCR | vegetation | no |  |
| (48) | Santos-Silva et al. 2017 | Portugal | D.m. (1/13), Hy.l. (31/175), I.r. (1/4), R.b. (0/7), R.p. (1/21), R.s. (0/11) | 231 | 34 | 0.14 (0.10-0.19) | qPCR (IS1111) | mixed | no |  |
| (49) | Satta et al. 2011 | Italy | D.m. (0/15), H.s. (1/25), Hy.m. (0/15), R.b. (0/90), R.p. (0/5), R.s. (5/1045), R.t. (1/290) | 1485 | 7 | 0.00 (0.00-0.01) ^1^ | PCR (*sodB*) | animals | no |  |
| (50) | Schabereiter-Gurtner et al. 2003 | Austria | I.r. (0/22) | 22 | 0 | 0.00^1^ | PCR (16S rRNA) | vegetation |  | I.r. |
| (51) | Schötta et al. 2017 | Austria | I. r. (0/554) | 554 | 0 | 0.00 | PCR-RLB (*htpAB*) | vegetation |  |  |
| (52) | Smetanova et al. 2006 | Slovakia | D.m. (0/16), D.r. (0/9), I.r. (2/327) | 352 | 2 | 0.01 (0.00-0.01) | PCR (*com1*) | mixed | no |  |
| (53) | Socolovschi et al. 2012 | France | D.m. (0/2), H.spp. (0/1), H.p. (0/1), Hy.spp. (0/1), Hy.r. (0/1), I.spp. (0/12), R.s. (1/96), R.t. (0/4) | 118 | 1 | 0.01 (0.00-0.03) | qPCR (IS30A, IS1111) | animals | no |  |
| (54) | Spitalska et al. 2002 | Slovakia | D.r. (0/1), H.c. (1/26), I.r. (1/106) | 133 | 2 | 0.02 (0.00-0.04) | PCR (*com1*), hemocyte test | vegetation | no |  |
| (55) | Spitalska and Kocianova 2003a | Slovakia/ Hungary | D.m. (3/43), D.r. (0/1), H.c. (1/26), H.i. (0/7), I.r. (2/158) | 263^2^ | 6 | 0.03 (0.01-0.05) | PCR (*com1*), RFLP, hemocyte test | vegetation | no |  |
| (56) | Spitalska and Kocianova 2003b | Slovakia | D.r. (0/5), H.c. (1/32), I.r. (1/141) | 178^2^ | 2 | 0.01 (0.00-0.03) | PCR (*com1*), hemocyte test | vegetation | no |  |
| (57) | Spitalska et al. 2006 | Slovakia | I.r. (0/92) | 92 | 0 | 0.00 | PCR (*com1*) | animals |  |  |
| (58) | Spitalska et al. 2018 | Slovakia | D.r. (7/334), H.i. (0/108), I.r. (8/163) | 605 | 15 | 0.02 (0.01-0.04) | qPCR (16S rRNA/ *groEL*) | vegetation | yes (33 samples) | D.r., H.i., I.r. |
| (59) | Sprong et al. 2012 | The Netherlands | D.r. (0/1), I.r. (6/2976) | 2977 | 5^3^ | 0.00 | qPCR (IS1111, *icd*, *com1*) | mixed |  |  |
| (60) | Sprong et al. 2019 | Great Britain | D.r. (0/113) | 113 | 0 | 0.00 | qPCR (*icd*/IS1111) | vegetation | no |  |
|  |  | Belgium | D.r. (1/513)^3^ | 513 | 0^4^ | 0.00 (0.00-0.01) | qPCR (*icd*/IS1111) | vegetation |  |  |
|  |  | Germany | D.r. (0/255) | 255 | 0 | 0.00 | qPCR (*icd*/IS1111) | vegetation |  |  |
|  |  | The Netherlands | D.r. (0/860) | 860 | 0 | 0.00 | qPCR (*icd*/IS1111) | vegetation |  |  |
| (61) | Subramanian et al. 2012 | Slovakia | I.r. (0/80) | 80 | 0 | 0.00 | qPCR (IS1111) | vegetation | no |  |
| (62) | Sukara et al. 2018 | Serbia | D.r. (0/47), H.c. (0/7), I.r. (0/64) | 118 | 0 | 0.00 | qPCR (*com1*) | animals |  |  |
| (63) | Szymanska-Czerwinska et al. 2013 | Poland | I.r. (191/1200) | 1200 | 191 | 0.16 (0.14-0.18) | qPCR (IS1111) | vegetation | no |  |
| (64) | Tijsse-Klasen et al. 2015 | Great Britain | D.r. (0/61), H.p. (0/99) | 160 | 0 | 0.00 | qPCR | vegetation |  |  |
| (65) | Tokarevich et al. 2019 | Bulgaria^5^ | D.spp. (0/7), D.r. (1/54), H.spp. (0/1), Hy.spp. (0/3), I.r. (0/10), R.spp. (0/10), R.b. (0/2), R.s. (0/12) | 99 | 1 | 0.01 (0-0.03) | PCR (*com1*, 16SrRNA) | vegetation | yes |  |
| (66) | Toledo et al. 2009 | Spain | A.l. (0/1), D.m. (25/348), H.h. (0/6), H.p. (0/1), Hy.l. (67/795), Hy.m. (0/13), I.r. (0/8), R.b. (0/56), R.p. (1/108), R.s. (2/146) | 1482 | 95 | 0.06 (0.05-0.08) | PCR, RLB (IS1111) | mixed | no |  |
| (67) | Toma et al. 2014 | Italy | A.spp. (0/5), Hy.spp. (2/8), Hy.m. (10/37), Hy.r. (29/71), I.spp. (1/6) | 127 | 42 | 0.33 (0.25-0.41) | qPCR (*icd*) | animals | no |  |
| (68) | Tomanovic et al. 2013 | Serbia | D.m. (0/15), D.r. (2/53), H.c. (10/35), H.p. (0/2), I.r. (17/27) | 132 | 29 | 0.22 (0.15-0.29) | PCR (*sodB*) | vegetation | yes |  |
| (69) | Varela-Castro et al. 2018 | Spain | D.m. (1/11), H.p. (5/23), H.s. (6/28), Hy.m. (0/1), R.b. (52/601), R.s. (3/5) | 669 | 67 | 0.10 (0.08-0.12) ^1^ | PCR (IS1111) | animals | yes  (5 samples) |  |
| (70) | Vila et al. 2019 | Spain | D.r., Hy.m., I.r., I.h., R.s. | 1600 | 0 | 0.00^1^ | PCR (16srRNA) | animals |  | I.h., I.r., R.s. |
| (71) | Wallménius et al. 2012 | Sweden | I.r. (0/786) | 786 | 0 | 0.00 | qPCR (*icd*) | mixed |  |  |
| (72) | Zhang et al. 2019 | Slovakia | D.m. (0/24), D.r. (0/24) | 48 | 0 | 0.00 | PCR (16srRNA) | vegetation |  | D.m., D.r. (*C.* spp.) |

A.l*. Amblyomma latum*; A.v. *Amblyomma variegatum*; D.m. *Dermacentor marginatus*; D.r. *Dermacentor reticulatus*; H.c. *Haemaphysalis concinna*; H.h. *Haemaphysalis hispanica*; H.i. *Haemaphysalis inermis*; H.p. *Haemaphysalis punctata*; H.pa. *Haemaphysalis parva*; H.s. *Haemaphysalis sulcata*; Hy ae. *Hyalomma aegyptium*; Hy.d. *Hyalomma dromedarii*; Hy.l. *Hyalomma lusitanicum*; Hy.m. *Hyalomma marginatum*; Hy.r. *Hyalomma rufipes*; Hy.s. *Hyalomma scupense*; Hy.t. *Hyalomma truncatum*; I.ar. *Ixodes arbicola*; I.c. *Ixodes crenulatus*; I.fe. *Ixodes festai*; I.fr. *Ixodes frontalis*; I.g. *Ixodes gibbosus*; I.h. *Ixodes hexagonus*; I.r. *Ixodes ricinus*; I.v. *Ixodes ventalloi*; R.a. *Rhipicephalus annulatus*; R.b. *Rhipicephalus bursa*; R.p. *Rhipicephalus pusillus*; R.r. *Rhipicephalus rossicus*; R.s. *Rhipicephalus sanguineus* s.l.; R.t. *Rhipicephalus turanicus*

N amount of ticks tested for *Coxiella*-DNA; n *Coxiella*-positive ticks

^1^Ticks tested in pools, MIR ^2^Different total amount given in the Text ^3^ sampled from vaccinated herd, 5 further ticks only IS1111 positive ^4^1 tick *icd* positive but IS1111 negative ^5^Further ticks were collected in Russia

1. Andersson MO, Tolf C, Tamba P, Stefanache M, Radbea G, Frangoulidis D, et al. Molecular survey of neglected bacterial pathogens reveals an abundant diversity of species and genotypes in ticks collected from animal hosts across Romania. Parasites & vectors (2018) 11(1):144. doi: 10.1186/s13071-018-2756-1

2. Astobiza I, Barral M, Ruiz-Fons F, Barandika JF, Gerrikagoitia X, Hurtado A, et al. Molecular investigation of the occurrence of *Coxiella burnetii* in wildlife and ticks in an endemic area. Vet Microbiol (2011) 147(1-2):190-4. doi: 10.1016/j.vetmic.2010.05.046

3. Barandika JF, Hurtado A, Garcia-Sanmartin J, Juste RA, Anda P, Garcia-Perez AL. Prevalence of tick-borne zoonotic bacteria in questing adult ticks from northern Spain. Vector Borne Zoonotic Dis (2008) 8(6):829-35. doi: 10.1089/vbz.2008.0023

4. Beltrame A, Laroche M, Degani M, Perandin F, Bisoffi Z, Raoult D, et al. Tick-borne pathogens in removed ticks Veneto, northeastern Italy: A cross-sectional investigation. Travel Medicine and Infectious Disease (2018) 26. doi: 10.1016/j.tmaid.2018.08.008

5. Bernasconi MV, Casati S, Peter O, Piffaretti JC. *Rhipicephalus* ticks infected with *Rickettsia* and *Coxiella* in Southern Switzerland (Canton Ticino). Infect Genet Evol (2002) 2(2):111-20. doi: 10.1016/s1567-1348(02)00092-8

6. Berthová L, Slobodník V, Slobodník R, Olekšák M, Sekeyová Z, Svitálková Z, et al. The natural infection of birds and ticks feeding on birds with *Rickettsia* spp. and *Coxiella burnetii* in Slovakia. Exp Appl Acarol (2016) 68(3):299-314. doi: 10.1007/s10493-015-9975-3

7. Bielawska-Drózd A, Cieślik P, Żakowska D, Głowacka P, Wlizło-Skowronek B, Zięba P, et al. Detection of *Coxiella burnetii* and *Francisella tularensis* in Tissues of Wild-living Animals and in Ticks of North-west Poland. Pol J Microbiol (2018) 67(4):529-34. doi: 10.21307/pjm-2018-059

8. Bogunovic D, Stević N, Sidi-Boumedine K, Misic D, Tomanović S, Kulišić Z, et al. Molecular evidence of Q Fever agent *Coxiella burnetii* in ixodid ticks collected from stray dogs in Belgrade (Serbia). Acta Veterinaria (2018) 68:257-68. doi: 10.2478/acve-2018-0023

9. Bonnet S, de la Fuente J, Nicollet P, Liu X, Madani N, Blanchard B, et al. Prevalence of tick-borne pathogens in adult *Dermacentor* spp. ticks from nine collection sites in France. Vector Borne Zoonotic Dis (2013) 13(4):226-36. doi: 10.1089/vbz.2011.0933

10. Cerutti F, Modesto P, Rizzo F, Cravero A, Jurman I, Costa S, et al. The microbiota of hematophagous ectoparasites collected from migratory birds. PLoS One (2018) 13(8):e0202270. doi: 10.1371/journal.pone.0202270

11. Chaligiannis I, Fernandez de Mera IG, Papa A, Sotiraki S, de la Fuente J. Molecular identification of tick-borne pathogens in ticks collected from dogs and small ruminants from Greece. Exp Appl Acarol (2018) 74(4):443-53. doi: 10.1007/s10493-018-0237-z

12. Chisu V, Foxi C, Mannu R, Satta G, Masala G. A five-year survey of tick species and identification of tick-borne bacteria in Sardinia, Italy. Ticks Tick Borne Dis (2018) 9(3):678-81. doi: 10.1016/j.ttbdis.2018.02.008

13. Chisu V, Loi F, Foxi C, Chessa G, Masu G, Rolesu S, et al. Coexistence of tick-borne pathogens in ticks collected from their hosts in Sardinia: an update. Acta Parasitol (2020). doi: 10.1007/s11686-020-00240-z

14. Chisu V, Foxi C, Masu G, B DA, Masala G. Detection of potentially pathogenic bacteria from *Ixodes ricinus* carried by pets in Tuscany, Italy. Vet Rec Open (2020) 7(1):e000395. doi: 10.1136/vetreco-2020-000395

15. Chitimia-Dobler L, Schaper S, Rieß R, Bitterwolf K, Frangoulidis D, Bestehorn M, et al. Imported *Hyalomma* ticks in Germany in 2018. Parasites & vectors (2019) 12(1):134. doi: 10.1186/s13071-019-3380-4

16. Desjardins I, Joulié A, Pradier S, Lecollinet S, Beck C, Vial L, et al. Seroprevalence of horses to *Coxiella burnetii* in an Q fever endemic area. Vet Microbiol (2018) 215:49-56. doi: 10.1016/j.vetmic.2017.11.012

17. Duh D, Slovák M, Saksida A, Strašek K, Petrovec M, Avšič-Županc T. Molecular detection of *Babesia canis* in *Dermacentor reticulatus* ticks collected in Slovakia. Biologia (2006) 61(2):231-3. doi: 10.2478/s11756-006-0035-7

18. Ebani VV, Bertelloni F, Turchi B, Filogari D, Cerri D. Molecular survey of tick-borne pathogens in Ixodid ticks collected from hunted wild animals in Tuscany, Italy. Asian Pac J Trop Med (2015) 8(9):714-7. doi: 10.1016/j.apjtm.2015.07.033

19. González J, González MG, Valcárcel F, Sánchez M, Martín-Hernández R, Tercero JM, et al. Prevalence of *Coxiella burnetii* (Legionellales: Coxiellaceae) infection among wildlife species and the tick *Hyalomma lusitanicum* (Acari: Ixodidae) in a meso-Mediterranean ecosystem. J Med Entomol (2020) 57(2):551-6. doi: 10.1093/jme/tjz169

20. Grech-Angelini S, Stachurski F, Vayssier-Taussat M, Devillers E, Casabianca F, Lancelot R, et al. Tick-borne pathogens in ticks (Acari: Ixodidae) collected from various domestic and wild hosts in Corsica (France), a Mediterranean island environment. Transbound Emerg Dis (2020) 67(2):745-57. doi: 10.1111/tbed.13393

21. Gyuranecz M, Dénes B, Hornok S, Kovács P, Horváth G, Jurkovich V, et al. Prevalence of *Coxiella burnetii* in Hungary: screening of dairy cows, sheep, commercial milk samples, and ticks. Vector Borne Zoonotic Dis (2012) 12(8):650-3. doi: 10.1089/vbz.2011.0953

22. Hartelt K, Pluta S, Oehme R, Kimmig P. Spread of ticks and tick-borne diseases in Germany due to global warming. Parasitol Res (2008) 103 Suppl 1:S109-16. doi: 10.1007/s00436-008-1059-4

23. Henning K, Greiner-Fischer S, Hotzel H, Ebsen M, Theegarten D. Isolation of *Spiroplasma* sp. from an *Ixodes* tick. Int J Med Microbiol (2006) 296 Suppl 40:157-61. doi: 10.1016/j.ijmm.2006.01.012

24. Hildebrandt A, Straube E, Neubauer H, Schmoock G. *Coxiella burnetii* and coinfections in *Ixodes ricinus* ticks in Central Germany. Vector Borne Zoonotic Dis (2011) 11(8):1205-7. doi: 10.1089/vbz.2010.0180

25. Hornok S, Csörgő T, de la Fuente J, Gyuranecz M, Privigyei C, Meli M, et al. Synanthropic Birds Associated with High Prevalence of Tick-Borne *Rickettsiae* and with the First Detection of *Rickettsia aeschlimannii* in Hungary. Vector Borne Zoonotic Dis (2013) 13. doi: 10.1089/vbz.2012.1032

26. Kalmár Z, Dumitrache M, d'amico G, Matei I, Ionică A, Gherman C, et al. Multiple tick-borne pathogens in *Ixodes ricinus* ticks collected from humans in Romania. Pathogens (2020) 9:390. doi: 10.3390/pathogens9050390

27. Kazimírová M, Hamšíková Z, Špitalská E, Minichová L, Mahríková L, Caban R, et al. Diverse tick-borne microorganisms identified in free-living ungulates in Slovakia. Parasites & vectors (2018) 11(1):495. doi: 10.1186/s13071-018-3068-1

28. Knap N, Zele D, Glinsek Biskup U, Avsic-Zupanc T, Vengust G. The prevalence of *Coxiella burnetii* in ticks and animals in Slovenia. BMC Vet Res (2019) 15(1):368. doi: 10.1186/s12917-019-2130-3

29. Mancini F, Di Luca M, Toma L, Vescio F, Bianchi R, Khoury C, et al. Prevalence of tick-borne pathogens in an urban park in Rome, Italy. Ann Agric Environ Med (2014) 21(4):723-7. doi: 10.5604/12321966.1129922

30. Mancini F, Vescio MF, Toma L, Di Luca M, Severini F, Caccio SM, et al. Detection of tick-borne pathogens in ticks collected in the suburban area of Monte Romano, Lazio Region, Central Italy. Ann Ist Super Sanita (2019) 55(2):143-50. doi: 10.4415/ann_19_02_06

31. Michelet L, Delannoy S, Devillers E, Umhang G, Aspan A, Juremalm M, et al. High-throughput screening of tick-borne pathogens in Europe. Front Cell Infect Microbiol (2014) 4:103. doi: 10.3389/fcimb.2014.00103

32. Michelet L, Joncour G, Devillers E, Torina A, Vayssier-Taussat M, Bonnet SI, et al. Tick species, tick-borne pathogens and symbionts in an insular environment off the coast of Western France. Ticks Tick Borne Dis (2016) 7(6):1109-15. doi: 10.1016/j.ttbdis.2016.08.014

33. Millán J, Proboste T, Fernández de Mera IG, Chirife AD, de la Fuente J, Altet L. Molecular detection of vector-borne pathogens in wild and domestic carnivores and their ticks at the human-wildlife interface. Ticks Tick Borne Dis (2016) 7(2):284-90. doi: 10.1016/j.ttbdis.2015.11.003

34. Minichová L, Hamšíková Z, Mahríková L, Slovák M, Kocianová E, Kazimírová M, et al. Molecular evidence of *Rickettsia* spp. in ixodid ticks and rodents in suburban, natural and rural habitats in Slovakia. Parasites & vectors (2017) 10(1):158. doi: 10.1186/s13071-017-2094-8

35. Pajoro M, Pistone D, Varotto Boccazzi I, Mereghetti V, Bandi C, Fabbi M, et al. Molecular screening for bacterial pathogens in ticks (*Ixodes ricinus*) collected on migratory birds captured in northern Italy. Folia Parasitol (Praha) (2018) 65. doi: 10.14411/fp.2018.008

36. Papa A, Tsioka K, Kontana A, Papadopoulos C, Giadinis N. Bacterial pathogens and endosymbionts in ticks. Ticks Tick Borne Dis (2017) 8(1):31-5. doi: 10.1016/j.ttbdis.2016.09.011

37. Pascucci I, Di Domenico M, Dall'Acqua F, Sozio G, Cammà C. Detection of Lyme disease and Q Fever agents in wild rodents in Central Italy. Vector Borne Zoonotic Dis (2015) 15(7):404-11. doi: 10.1089/vbz.2015.1807

38. Pascucci I, Di Domenico M, Capobianco Dondona G, Di Gennaro A, Polci A, Capobianco Dondona A, et al. Assessing the role of migratory birds in the introduction of ticks and tick-borne pathogens from African countries: An Italian experience. Ticks Tick Borne Dis (2019) 10(6):101272. doi: 10.1016/j.ttbdis.2019.101272

39. Paștiu AI, Matei IA, Mihalca AD, D’Amico G, Dumitrache MO, Kalmár Z, et al. Zoonotic pathogens associated with *Hyalomma aegyptium* in endangered tortoises: evidence for host-switching behaviour in ticks? Parasites & vectors (2012) 5(1):301. doi: 10.1186/1756-3305-5-301

40. Pilloux L, Baumgartner A, Jaton K, Lienhard R, Ackermann-Gaumann R, Beuret C, et al. Prevalence of *Anaplasma phagocytophilum* and *Coxiella burnetii* in *Ixodes ricinus* ticks in Switzerland: an underestimated epidemiologic risk. New Microbes New Infect (2019) 27:22-6. doi: 10.1016/j.nmni.2018.08.017

41. Pistone D, Pajoro M, Novakova E, Vicari N, Gaiardelli C, Viganò R, et al. Ticks and bacterial tick-borne pathogens in Piemonte region, Northwest Italy. Exp Appl Acarol (2017) 73(3-4):477-91. doi: 10.1007/s10493-017-0202-2

42. Pluta S, Hartelt K, Oehme R, Mackenstedt U, Kimmig P. Prevalence of *Coxiella burnetii* and *Rickettsia* spp. in ticks and rodents in southern Germany. Ticks Tick Borne Dis (2010) 1(3):145-7. doi: 10.1016/j.ttbdis.2010.04.001

43. Quarsten H, Skarpaas T, Fajs L, Noraas S, Kjelland V. Tick-borne bacteria in *Ixodes ricinus* collected in southern Norway evaluated by a commercial kit and established real-time PCR protocols. Ticks Tick Borne Dis (2015) 6(4):538-44. doi: 10.1016/j.ttbdis.2015.04.008

44. Raele DA, Galante D, Pugliese N, De Simone E, Cafiero MA. *Coxiella*-like endosymbiont associated to the "Anatolian brown tick" *Rhipicephalus bursa* in Southern Italy. Microbes Infect (2015) 17(11-12):799-805. doi: 10.1016/j.micinf.2015.09.011

45. Reye AL, Hubschen JM, Sausy A, Muller CP. Prevalence and seasonality of tick-borne pathogens in questing *Ixodes ricinus* ticks from Luxembourg. Appl Environ Microbiol (2010) 76(9):2923-31. doi: 10.1128/aem.03061-09

46. Reye AL, Stegniy V, Mishaeva NP, Velhin S, Hubschen JM, Ignatyev G, et al. Prevalence of tick-borne pathogens in *Ixodes ricinus* and *Dermacentor reticulatus* ticks from different geographical locations in Belarus. PLoS One (2013) 8(1):e54476. doi: 10.1371/journal.pone.0054476

47. Santos AS, de Bruin A, Veloso AR, Marques C, Pereira da Fonseca I, de Sousa R, et al. Detection of *Anaplasma phagocytophilum*, *Candidatus* Neoehrlichia sp., *Coxiella burnetii* and *Rickettsia* spp. in questing ticks from a recreational park, Portugal. Ticks Tick Borne Dis (2018) 9(6):1555-64. doi: 10.1016/j.ttbdis.2018.07.010

48. Santos-Silva MM, Melo P, Santos N, Antunes S, Duarte LR, Ferrolho J, et al. PCR screening of tick-borne agents in sensitive conservation areas, Southeast Portugal. Mol Cell Probes (2017) 31:42-5. doi: 10.1016/j.mcp.2016.11.005

49. Satta G, Chisu V, Cabras P, Fois F, Masala G. Pathogens and symbionts in ticks: a survey on tick species distribution and presence of tick-transmitted micro-organisms in Sardinia, Italy. J Med Microbiol (2011) 60(Pt 1):63-8. doi: 10.1099/jmm.0.021543-0

50. Schabereiter-Gurtner C, Lubitz W, Rölleke S. Application of broad-range 16S rRNA PCR amplification and DGGE fingerprinting for detection of tick-infecting bacteria. J Microbiol Methods (2003) 52(2):251-60. doi: 10.1016/s0167-7012(02)00186-0

51. Schötta AM, Wijnveld M, Stockinger H, Stanek G. Approaches for Reverse Line Blot-Based Detection of Microbial Pathogens in *Ixodes ricinus* Ticks Collected in Austria and Impact of the Chosen Method. Appl Environ Microbiol (2017) 83(13). doi: 10.1128/aem.00489-17

52. Smetanová K, Schwarzová K, Kocianová E. Detection of *Anaplasma phagocytophilum*, *Coxiella burnetii*, *Rickettsia* spp., and *Borrelia burgdorferi* s. l. in ticks, and wild-living animals in western and middle Slovakia. Ann N Y Acad Sci (2006) 1078:312-5. doi: 10.1196/annals.1374.058

53. Socolovschi C, Reynaud P, Kernif T, Raoult D, Parola P. Rickettsiae of spotted fever group, *Borrelia valaisiana*, and *Coxiella burnetii* in ticks on passerine birds and mammals from the Camargue in the south of France. Ticks Tick Borne Dis (2012) 3(5-6):355-60. doi: 10.1016/j.ttbdis.2012.10.019

54. Spitalská E, Kocianová E, Výrosteková V. Natural focus of *Coxiella burnetii* and *Rickettsiae* of spotted fever group in southwestern Slovakia. Biologia (2002) 57:585-91. doi:

55. Spitalska E, Kocianova E. Detection of *Coxiella burnetii* in ticks collected in Slovakia and Hungary. Eur J Epidemiol (2003) 18(3):263-6. doi: 10.1023/a:1023330222657

56. Spitalska E, Kocianova E. Tick-borne microorganisms in southwestern Slovakia. Ann N Y Acad Sci (2003) 990:196-200. doi: 10.1111/j.1749-6632.2003.tb07362.x

57. Spitalska E, Literak I, Sparagano OA, Golovchenko M, Kocianova E. Ticks (Ixodidae) from passerine birds in the Carpathian region. Wien Klin Wochenschr (2006) 118(23-24):759-64. doi: 10.1007/s00508-006-0729-4

58. Spitalska E, Sparagano O, Stanko M, Schwarzova K, Spitalsky Z, Skultety L, et al. Diversity of *Coxiella*-like and *Francisella*-like endosymbionts, and *Rickettsia* spp., *Coxiella burnetii* as pathogens in the tick populations of Slovakia, Central Europe. Ticks Tick Borne Dis (2018) 9(5):1207-11. doi: 10.1016/j.ttbdis.2018.05.002

59. Sprong H, Tijsse-Klasen E, Langelaar M, De Bruin A, Fonville M, Gassner F, et al. Prevalence of *Coxiella burnetii* in ticks after a large outbreak of Q fever. Zoonoses Public Health (2012) 59(1):69-75. doi: 10.1111/j.1863-2378.2011.01421.x

60. Sprong H, Fonville M, Docters van Leeuwen A, Devillers E, Ibañez-Justicia A, Stroo A, et al. Detection of pathogens in *Dermacentor reticulatus* in northwestern Europe: evaluation of a high-throughput array. Heliyon (2019) 5(2):e01270. doi: 10.1016/j.heliyon.2019.e01270

61. Subramanian G, Sekeyova Z, Raoult D, Mediannikov O. Multiple tick-associated bacteria in *Ixodes ricinus* from Slovakia. Ticks Tick Borne Dis (2012) 3(5-6):406-10. doi: 10.1016/j.ttbdis.2012.10.001

62. Sukara R, Chochlakis D, Ćirović D, Penezić A, Mihaljica D, Ćakić S, et al. Golden jackals (*Canis aureus*) as hosts for ticks and tick-borne pathogens in Serbia. Ticks Tick Borne Dis (2018) 9(5):1090-7. doi: 10.1016/j.ttbdis.2018.04.003

63. Szymanska-Czerwinska M, Galinska EM, Niemczuk K, Zasepa M. Prevalence of *Coxiella burnetii* infection in foresters and ticks in the south-eastern Poland and comparison of diagnostic methods. Ann Agric Environ Med (2013) 20(4):699-704. doi:

64. Tijsse-Klasen E, Hansford KM, Jahfari S, Phipps P, Sprong H, Medlock JM. Spotted fever group rickettsiae in *Dermacentor reticulatus* and *Haemaphysalis punctata* ticks in the UK. Parasites & vectors (2013) 6:212. doi: 10.1186/1756-3305-6-212

65. Tokarevich NK, Panferova YA, Freylikhman OA, Blinova OV, Medvedev SG, Mironov SV, et al. *Coxiella burnetii* in ticks and wild birds. Ticks Tick Borne Dis (2019) 10(2):377-85. doi: 10.1016/j.ttbdis.2018.11.020

66. Toledo A, Jado I, Olmeda AS, Casado-Nistal MA, Gil H, Escudero R, et al. Detection of *Coxiella burnetii* in ticks collected from Central Spain. Vector Borne Zoonotic Dis (2009) 9(5):465-8. doi: 10.1089/vbz.2008.0070

67. Toma L, Mancini F, Di Luca M, Cecere JG, Bianchi R, Khoury C, et al. Detection of microbial agents in ticks collected from migratory birds in central Italy. Vector Borne Zoonotic Dis (2014) 14(3):199-205. doi: 10.1089/vbz.2013.1458

68. Tomanovic S, Chochlakis D, Radulovic Z, Milutinovic M, Cakic S, Mihaljica D, et al. Analysis of pathogen co-occurrence in host-seeking adult hard ticks from Serbia. Exp Appl Acarol (2013) 59(3):367-76. doi: 10.1007/s10493-012-9597-y

69. Varela-Castro L, Zuddas C, Ortega N, Serrano E, Salinas J, Castellà J, et al. On the possible role of ticks in the eco-epidemiology of *Coxiella burnetii* in a Mediterranean ecosystem. Ticks Tick Borne Dis (2018) 9(3):687-94. doi: 10.1016/j.ttbdis.2018.02.014

70. Vila A, Estrada-Peña A, Altet L, Cusco A, Dandreano S, Francino O, et al. Endosymbionts carried by ticks feeding on dogs in Spain. Ticks Tick Borne Dis (2019) 10(4):848-52. doi: https://doi.org/10.1016/j.ttbdis.2019.04.003

71. Wallmenius K, Pettersson JH, Jaenson TG, Nilsson K. Prevalence of *Rickettsia* spp., *Anaplasma phagocytophilum*, and *Coxiella burnetii* in adult *Ixodes ricinus* ticks from 29 study areas in central and southern Sweden. Ticks Tick Borne Dis (2012) 3(2):100-6. doi: 10.1016/j.ttbdis.2011.11.003

72. Zhang Y-K, Yu Z-J, Wang D, Bronislava V, Branislav P, Liu J-Z. The bacterial microbiome of field-collected *Dermacentor marginatus* and *Dermacentor reticulatus* from Slovakia. Parasites & vectors (2019) 12(1):325. doi: 10.1186/s13071-019-3582-9
